# Identification of prognostic and predictive biomarkers in high-dimensional data with PPLasso

**DOI:** 10.1186/s12859-023-05143-0

**Published:** 2023-01-23

**Authors:** Wencan Zhu, Céline Lévy-Leduc, Nils Ternès

**Affiliations:** 1 Université Paris-Saclay, AgroParisTech, INRAE, UMR MIA Paris-Saclay, 91120 Palaiseau, France; 2 Biostatistics and Programming Department, Sanofi R&D, 91380 Chilly Mazarin, France

**Keywords:** Variable selection, Highly correlated predictors, Genomic data

## Abstract

In clinical trials, identification of prognostic and predictive biomarkers has became essential to precision medicine. Prognostic biomarkers can be useful for the prevention of the occurrence of the disease, and predictive biomarkers can be used to identify patients with potential benefit from the treatment. Previous researches were mainly focused on clinical characteristics, and the use of genomic data in such an area is hardly studied. A new method is required to simultaneously select prognostic and predictive biomarkers in high dimensional genomic data where biomarkers are highly correlated. We propose a novel approach called PPLasso, that integrates prognostic and predictive effects into one statistical model. PPLasso also takes into account the correlations between biomarkers that can alter the biomarker selection accuracy. Our method consists in transforming the design matrix to remove the correlations between the biomarkers before applying the generalized Lasso. In a comprehensive numerical evaluation, we show that PPLasso outperforms the traditional Lasso and other extensions on both prognostic and predictive biomarker identification in various scenarios. Finally, our method is applied to publicly available transcriptomic and proteomic data.

**Supplementary Information:**

The online version contains supplementary material available at 10.1186/s12859-023-05143-0.

## Introduction

With the development of precision medicine, there has been an increasing interest in the discovery of different types of biomarkers. A prognostic biomarker informs about a likely clinical outcome (e.g., disease recurrence, disease progression, death) in the absence of therapy or with a standard therapy that patients are likely to receive, while a predictive biomarker is associated with a response or a lack of response to a specific therapy. Fourati [3] and Clark [8] provided a comprehensive explanation and concrete examples to distinguish prognostic from predictive biomarkers, respectively. During the past decade, prognostic and predictive biomarkers showed their power in the development of precision medicine. Giannos et al. [15] identified ten prognostic gene biomarkers for non-small cell lung cancer that can be useful for improving risk prediction and therapeutic strategies. Zhao et al. [38] obtained prognostic biomarkers for 13 cancers by integrating multi-omics data and provided a reference for translational medicine researchers. He et al. [16] identified predictive biomarkers for sorafenib resistance and contributed to the guidance of individualized drug therapy. Yet, correctly identifying such biomarkers remains difficult.

Concerning the biomarker selection, the high dimensionality of genomic data is one of the main challenges as explained in Fan and Li [9]. To identify effective biomarkers in high-dimensional settings, several approaches can be considered including hypothesis-based tests described in [23], wrapper approaches proposed in [25], and penalized approaches such as Lasso designed by [30] among others. Hypothesis-based tests consider each biomarker independently and thus ignore potential correlations between them. Wrapper approaches often show high risk of overfitting and are computationally expensive for high-dimensional data as explained in [27]. More efforts have been devoted to penalized methods given their ability to automatically perform variable selection and coefficient estimation simultaneously as highlighted in [10]. However, Lasso showed some potential drawbacks when biomarkers are highly correlated. Particularly, when the Irrepresentable Condition (IC) proposed by [39] is violated, Lasso can not guarantee to correctly identify true effective biomarkers. In genomic data, biomarkers are usually highly correlated such that this condition can hardly be satisfied, see [34]. Several methods have been proposed to adress this issue. Elastic Net [41] combines the $\ell _1$ and $\ell _2$ penalties and is particularly effective in tackling correlation issues and can generally outperform Lasso. Adaptive Lasso [42] proposes to assign adaptive weights for penalizing different coefficients in the $\ell _1$ penalty, and its oracle property was demonstrated. Wang and Leng [35] proposed the HOLP approach which consists in removing the correlation between the columns of the design matrix; Wang et al. [34] proposed to handle the correlation by assigning similar weights to correlated variables in their approach called Precision Lasso; Zhu et al. [40] proposed to remove the correlations by applying a whitening transformation to the data before using the generalized Lasso criterion designed by [31].

The challenge of finding prognostic biomarkers has been extensively explored with previously introduced methods, however, the discovery of predictive biomarkers has seen much less attention. Limited to binary endpoint, Foster et al. [13] proposed to first predict response probabilities for treatment and use this probability as the response in a classification problem to find effective biomarkers. Tian et al. [29] proposed a new method to detect interaction between the treatment and the biomarkers by modifying the covariates. This method can be implemented on continuous/binary/time-to-event endpoint. Lipkovich et al. [20] proposed a method called SIDES, which adopts a recursive partitioning algorithm for screening treatment-by-biomarker interactions. This method was further improved in [19] by adding another step of preselection on predictive biomarkers based on variable importance. The method was demonstrated with continuous endpoint. Evaluated on time-to-event data, Ternès et al.[28] proposed a framework for identifying biomarker-by-treatment interactions but not specifically in the context of correlated biomarkers. More recently, Sechidis et al. [26] applied approaches coming from information theory for ranking biomarkers on their prognostic/predictive strength. Their method is applicable only for binary or time-to-event endpoint. Moreover, all of these methods were assessed under the situation where the sample size is relatively large and the number of biomarkers is limited, which is hardly the case for genomic data.

In the literature mentioned above, the authors focused on one of the problematic of identifying prognostic or predictive biomarkers, but rarely on both. Even if predictive biomarkers is of major importance for identifying patients more likely to benefit from a treatment, the prognostic biomarkers is also key in this context. Indeed, the clinical impact of a treatment can be judged only with the knowledge of the prognosis of a patient. It is thus of importance to reliably predict the prognosis of patients to assist treatment counseling [36]. To properly describe the two effects, the experimental treatment should be compared to a standard therapy (or a placebo), and patients receiving different treatments should be randomized. A randomized clinical trial can be ideal for such a study. In this paper, we developed a new method called PPLasso (Prognostic Predictive Lasso) to identify prognostic and at the same time predictive biomarkers in a high dimensional setting with continuous endpoints, as presented in “Methods” section . Extensive numerical experiments are given in “Numerical experiments” section  to assess the performance of our approach and to compare it to other methods. PPLasso is also applied on two publicly available transcriptomic and proteomic data in “Application to transcriptomic and proteomic data” section. Finally, we give concluding remarks in “Conclusion” section .

## Methods

In this section, we propose a novel approach called PPLasso (Predictive Prognostic Lasso) which consists in writing the identification of predictive and prognostic biomarkers as a variable selection problem in an ANCOVA (Analysis of Covariance) type model mentioned for instance in [12].

### Statistical modeling

Let ${\textbf{y}}$ be a continuous response or endpoint and $t_1$, $t_2$ two treatments. Let also ${\textbf{X}}_{1}$ (resp. ${\textbf{X}}_{2}$) denote the design matrix for the $n_1$ (resp. $n_2$) patients with treatment $t_1$ (resp. $t_{2}$), each containing measurements on *p* candidate biomarkers:

1 $$\begin{aligned} {\textbf{X}}_1= \begin{bmatrix} X_{11}^{1} &{} X_{11}^{2} &{} \ldots &{} X_{11}^{p} \\ X_{12}^{1} &{} X_{12}^{2} &{} \ldots &{} X_{12}^{p} \\ ...\\ X_{1n_{1}}^{1} &{} X_{1n_{1}}^{2} &{} \ldots &{} X_{1n_{1}}^{p} \end{bmatrix},{\textbf{X}}_2=\begin{bmatrix} X_{21}^{1} &{} X_{21}^{2} &{} \ldots &{} X_{21}^{p} \\ X_{22}^{1} &{} X_{22}^{2} &{} \ldots &{} X_{22}^{p} \\ ...\\ X_{2n_{2}}^{1} &{} X_{2n_{2}}^{2} &{} \ldots &{} X_{2n_{2}}^{p} \end{bmatrix}. \end{aligned}$$

To take into account the potential correlation that may exist between the biomarkers in the different treatments, we shall assume that the rows of ${\textbf{X}}_{1}$ (resp. ${\textbf{X}}_{2}$) are independent centered Gaussian random vectors with a covariance matrice equal to $\varvec{\Sigma _1}$ (resp. $\varvec{\Sigma _2}$).

To model the link that exists between ${\textbf{y}}$ and the different types of biomarkers we propose using the following model:

2 $$\begin{aligned} {\textbf{y}}=\begin{pmatrix} y_{11} \\ y_{12}\\ \vdots \\ y_{1n_{1}}\\ y_{21} \\ y_{22}\\ \vdots \\ y_{2n_{2}} \end{pmatrix} ={\textbf{X}}\begin{pmatrix} \alpha _1 \\ \alpha _2 \\ \beta _{11} \\ \beta _{12}\\ \vdots \\ \beta _{1p} \\ \beta _{21} \\ \beta _{22}\\ \vdots \\ \beta _{2p} \end{pmatrix} + \begin{pmatrix} \epsilon _{11} \\ \epsilon _{12}\\ \vdots \\ \epsilon _{1n_{1}}\\ \epsilon _{21} \\ \epsilon _{22}\\ \vdots \\ \epsilon _{2n_{2}} \end{pmatrix}, \end{aligned}$$

where $(y_{i1},\dots ,y_{in_i})$ corresponds to the response of patients with treatment $t_i$, *i* being equal to 1 or 2,

$$\begin{aligned} {\textbf{X}}=\begin{bmatrix} 1 &{} 0 &{} X_{11}^{1} &{} X_{11}^{2} &{} \ldots &{} X_{11}^{p} &{} 0 &{} 0 &{} \ldots &{} 0 \\ 1 &{} 0 &{} X_{12}^{1} &{} X_{12}^{2} &{} \ldots &{} X_{12}^{p} &{} 0 &{} 0 &{} \ldots &{} 0 \\ \vdots &{} \vdots &{} \vdots &{} \vdots &{} &{} \vdots &{} &{} &{} \\ 1 &{} 0 &{} X_{1n_{1}}^{1} &{} X_{1n_{1}}^{2} &{} \ldots &{} X_{1n_{1}}^{p} &{} 0 &{} 0 &{} \ldots &{} 0 \\ 0 &{} 1 &{} 0 &{} 0 &{} \ldots &{} 0 &{} X_{21}^{1} &{} X_{21}^{2} &{} \ldots &{} X_{21}^{p} \\ 0 &{} 1 &{} 0 &{} 0 &{} \ldots &{} 0 &{} X_{22}^{1} &{} X_{22}^{2} &{} \ldots &{} X_{22}^{p} \\ \vdots &{} \vdots &{} \vdots &{} \vdots &{} &{} \vdots &{} \vdots &{}\vdots &{} &{}\vdots \\ 0 &{} 1 &{} 0 &{} 0 &{} \ldots &{} 0 &{} X_{2n_{2}}^{1} &{} X_{2n_{2}}^{2} &{} \ldots &{} X_{2n_{2}}^{p} \end{bmatrix}, \end{aligned}$$

with $\alpha _1$ (resp. $\alpha _2$) corresponding to the effects of treatment $t_1$ (resp. $t_2$). Moreover, $\varvec{\beta }_1=(\beta _{11}, \beta _{12}, \ldots , \beta _{1p})'$ (resp. $\varvec{\beta }_2=(\beta _{21}, \beta _{22}, \ldots , \beta _{2p})'$) are the coefficients associated to each of the *p* biomarkers in treatment $t_1$ (resp. $t_2$) group, $'$ denoting the matrix transposition and $\epsilon _{11},\dots ,\epsilon _{2n_2}$ are standard independent Gaussian random variables independent of ${\textbf{X}}_{1}$ and ${\textbf{X}}_{2}$. When $t_1$ stands for the standard treatment or placebo, prognostic biomarkers are defined as those having non-zero coefficients in $\varvec{\beta }_{1}$. According to the definition of prognostic biomarkers, their effect should indeed be demonstrated in the absence of therapy or with a standard therapy that patients are likely to receive. On the other hand, predictive biomarkers are defined as those having non-zero coefficients in $\varvec{\beta }_{2}-\varvec{\beta }_{1}$ because they aim to highlight different effects between two different treatments.

Model (2) can be written as:

3 $$\begin{aligned} {\textbf{y}}={\textbf{X}}\varvec{\gamma }+\varvec{\epsilon }, \end{aligned}$$

with $\varvec{\gamma }=(\alpha _1,\alpha _2, \varvec{\beta }_{1}', \varvec{\beta }_{2}')'$. The Lasso penalty is a well-known approach to estimate coefficients with a sparsity enforcing constraint allowing variable selection by estimating some coefficients by zero. It consists in minimizing the following penalized least-squares criterion [30]:

4 $$\begin{aligned} \frac{1}{2}\left\Vert {\textbf{y}}-{\textbf{X}}\varvec{\gamma }\right\Vert _{2}^{2}+\lambda \left\Vert \varvec{\gamma }\right\Vert _{1}, \end{aligned}$$

where $\left\Vert {{\textbf {u}}}\right\Vert _{2}^2=\sum _{i=1}^n u_i^2$ and $\left\Vert {{\textbf {u}}}\right\Vert _{1}=\sum _{i=1}^n |u_i|$ for ${{\textbf {u}}}=(u_1,\dots ,u_n)$. A different sparsity constraint was applied to $\varvec{\beta }_1$ and $\varvec{\beta }_{2}-\varvec{\beta }_{1}$ to allow different sparsity levels. Hence we propose to replace the penalty $\lambda \left\Vert \varvec{\gamma }\right\Vert _{1}$ in (4) by

5 $$\begin{aligned} \lambda _{1}\left\Vert \varvec{\beta }_{1}\right\Vert _{1}+\lambda _{2}\left\Vert \varvec{\beta }_{2}-\varvec{\beta }_{1}\right\Vert _{1}. \end{aligned}$$

Thus, a first estimator of $\varvec{\gamma }$ could be found by minimizing the following criterion with respect to $\varvec{\gamma }$:

6 $$\begin{aligned} \frac{1}{2}\left\Vert {\textbf{y}}-{\textbf{X}}\varvec{\gamma }\right\Vert _{2}^{2}+\lambda _{1}\left\Vert \begin{bmatrix} {\textbf{0}}_{p,1} &{} {\textbf{0}}_{p,1} &{} D_{1}\\ {\textbf{0}}_{p,1} &{} {\textbf{0}}_{p,1} &{} \frac{\lambda _{2}}{\lambda _{1}}D_{2}\end{bmatrix}\varvec{\gamma }\right\Vert _{1}, \end{aligned}$$

where $D_1=[{\text {Id}}_{p}, {\textbf{0}}_{p,p}]$ and $D_2=[-{\text {Id}}_{p},{\text {Id}}_{p}]$, with ${\text {Id}}_{p}$ denoting the identity matrix of size *p* and ${\textbf{0}}_{i,j}$ denoting a matrix having *i* rows and *j* columns and containing only zeros. However, since the inconsistency of Lasso biomarker selection is originated from the correlations between the biomarkers, we propose to remove the correlation by “whitening” the matrix ${\textbf{X}}$. More precisely, we consider ${\widetilde{{\textbf{X}}}}={\textbf{X}}\varvec{\Sigma }^{-1/2}$, where

7 $$\begin{aligned} \varvec{\Sigma }= \begin{bmatrix} 1 &{} 0 &{} 0 &{} 0\\ 0 &{} 1 &{} 0 &{} 0 \\ 0 &{} 0 &{} \varvec{\Sigma }_{1} &{} 0 \\ 0 &{} 0 &{} 0&{} \varvec{\Sigma }_{2} \end{bmatrix} \end{aligned}$$

and define $\varvec{\Sigma }^{-1/2}$ by replacing in (7) $\varvec{\Sigma }_{i}$ by $\varvec{\Sigma }_{i}^{-1/2}$, where $\varvec{\Sigma }_{i}^{-1/2}={\textbf{U}}_{i}{\textbf{D}}_{i}^{-1/2}{\textbf{U}}_{i}^{T}$, ${\textbf{U}}_{i}$ and ${\textbf{D}}_{i}$ being the matrices involved in the spectral decomposition of $\varvec{\Sigma }_{i}$ for $i=1$ or 2. With such a transformation the columns of ${\widetilde{{\textbf{X}}}}$ are decorrelated and Model (3) can be rewritten as follows:

8 $$\begin{aligned} {\textbf{y}}= {\widetilde{{\textbf{X}}}}{\widetilde{\varvec{\gamma }}}+\varvec{\epsilon }\end{aligned}$$

where ${\widetilde{\varvec{\gamma }}}=\varvec{\Sigma }^{1/2}\varvec{\gamma }$. The objective function (6) thus becomes:

9 $$\begin{aligned} L_{\lambda _1,\lambda _2}^{\text {PPLasso}}({\widetilde{\varvec{\gamma }}})=\frac{1}{2}\left\Vert {\textbf{y}}-{\widetilde{{\textbf{X}}}}{\widetilde{\varvec{\gamma }}}\right\Vert _{2}^{2}+\lambda _{1}\left\Vert \begin{bmatrix} {\textbf{0}}_{p,1} &{} {\textbf{0}}_{p,1} &{} D_{1}\\ {\textbf{0}}_{p,1} &{} {\textbf{0}}_{p,1} &{} \frac{\lambda _{2}}{\lambda _{1}}D_{2}\end{bmatrix}\varvec{\Sigma }^{-1/2}{\widetilde{\varvec{\gamma }}}\right\Vert _{1}. \end{aligned}$$

### Estimation of ${\widetilde{\varvec{\gamma }}}$

Let us define a first estimator of ${\widetilde{\varvec{\gamma }}}=({\widetilde{\alpha }}_1,{\widetilde{\alpha }}_2,{\widetilde{\varvec{\beta }}}_1',{\widetilde{\varvec{\beta }}}_2')$ as follows:

10 $$\begin{aligned} \widehat{{\widetilde{\varvec{\gamma }}}}_{0}(\lambda _1,\lambda _2)=(\widehat{{\widetilde{\alpha }}}_{1},\widehat{{\widetilde{\alpha }}}_{2}, \widehat{{\widetilde{\varvec{\beta }}}}_{10}', \widehat{{\widetilde{\varvec{\beta }}}}_{20}')=\mathop {\textrm{Argmin}}\nolimits _{{\widetilde{\varvec{\gamma }}}} L_{\lambda _1,\lambda _2}^{\text {PPLasso}}({\widetilde{\varvec{\gamma }}}), \end{aligned}$$

for each fixed $\lambda _1$ and $\lambda _2$. To better estimate ${\widetilde{\varvec{\beta }}}_1$ and ${\widetilde{\varvec{\beta }}}_2$, a thresholding was applied to $\widehat{{\widetilde{\varvec{\beta }}}}_{0}(\lambda _1,\lambda _2)=(\widehat{{\widetilde{\varvec{\beta }}}}_{10}(\lambda _1,\lambda _2)',\widehat{{\widetilde{\varvec{\beta }}}}_{20}(\lambda _1,\lambda _2)')'$. For $K_1$ (resp. $K_2$) in $\{1,\ldots ,p\}$, let $\text {Top}_{K_1}$ (resp. $\text {Top}_{K_2}$) be the set of indices corresponding to the $K_1$ (resp. $K_2$) largest values of the components of $|\widehat{{\widetilde{\varvec{\beta }}}}_{10}(\lambda _1,\lambda _2)|$ (resp. $|\widehat{{\widetilde{\varvec{\beta }}}}_{20}(\lambda _1,\lambda _2)|$), then the estimator of ${\widetilde{\varvec{\beta }}}=({\widetilde{\varvec{\beta }}}_1',{\widetilde{\varvec{\beta }}}_2')$ after the correction is denoted by $\widehat{{\widetilde{\varvec{\beta }}}}(\lambda _1,\lambda _2)=(\widehat{{\widetilde{\varvec{\beta }}}}_{1}^{({\widehat{K}}_1)}(\lambda _1,\lambda _2), \widehat{{\widetilde{\varvec{\beta }}}}_{2}^{({\widehat{K}}_2)}(\lambda _1,\lambda _2))$ where the *j*th component of $\widehat{{\widetilde{\varvec{\beta }}}}_{i}^{(K_i)}(\lambda _1,\lambda _2)$, for $i=1$ or 2, is defined by:

11 $$\begin{aligned} \widehat{{\widetilde{\varvec{\beta }}}}_{ij}^{(K_i)}(\lambda _{1}, \lambda _{2})= {\left\{ \begin{array}{ll} \widehat{{\widetilde{\varvec{\beta }}}}_{i0j}(\lambda _{1}, \lambda _{2}), &{} j \in \text {Top}_{K_i} \\ K_1\text {th largest value of } |\widehat{{\widetilde{\varvec{\beta }}}}_{i0j}(\lambda _{1}, \lambda _{2})| , &{} j \not \in \text {Top}_{K_i}. \end{array}\right. } \end{aligned}$$

Note that the corrections are only performed on $\widehat{{\widetilde{\varvec{\beta }}}}_0$, the estimators $\widehat{{\widetilde{\alpha }}}_{1}$ and $\widehat{{\widetilde{\alpha }}}_{2}$ were not modified. The choice of $K_1$ and $K_2$ will be explained in “[Sec Sec6]” section.

**Fig. 1:**
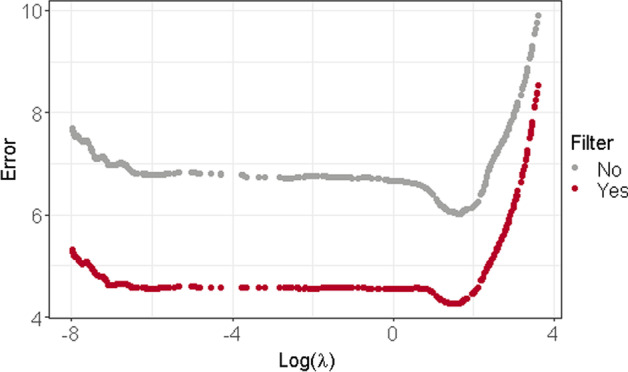
Estimation error $\left\Vert \widehat{{\widetilde{\varvec{\beta }}}}_{0}(\lambda )-{\widetilde{\varvec{\beta }}}\right\Vert _{2}$ (gray) and $\left\Vert \widehat{{\widetilde{\varvec{\beta }}}}(\lambda )-{\widetilde{\varvec{\beta }}}\right\Vert _{2}$ (red) for all $\lambda$

To illustrate the interest of using a thresholding step, we generated a dataset based on Model 3 with parameters described in “Simulation setting” section  and $p=500$. Moreover, to simplify the graphical illustrations, we focus on the case where $\lambda _{1}=\lambda _{2}=\lambda$. Figure 1 displays the estimation error associated to the estimators of ${\widetilde{\varvec{\beta }}}(\lambda )$ before and after the thresholding. We can see from this figure that the estimation of ${\widetilde{\varvec{\beta }}}(\lambda )$ is less biased after the correction. Moreover, we observed that this thresholding strongly improves the final estimation of $\varvec{\gamma }$ and the variable selection performance of our method.

### Estimation of $\varvec{\gamma }$

With $\widehat{{\widetilde{\varvec{\beta }}}} = (\widehat{{\widetilde{\varvec{\beta }}}}_{1}', \widehat{{\widetilde{\varvec{\beta }}}}_{2}')$, the estimators of $\varvec{\beta }_1$ and $\varvec{\beta }_2-\varvec{\beta }_1$ can be obtained by ${\widehat{\varvec{\beta }}}_{10}=\varvec{\Sigma }_{1}^{-1/2}\widehat{{\widetilde{\varvec{\beta }}}}_{1}$ and $({\widehat{\varvec{\beta }}}_{20}-{\widehat{\varvec{\beta }}}_{10})=\varvec{\Sigma }_{2}^{-1/2}\widehat{{\widetilde{\varvec{\beta }}}}_{2}-\varvec{\Sigma }_{1}^{-1/2}\widehat{{\widetilde{\varvec{\beta }}}}_{1}$. As previously, another thresholding was applied to ${\widehat{\varvec{\beta }}}_{10}$ and ${\widehat{\varvec{\beta }}}_{20}$: for $i=1$ or 2,

12 $$\begin{aligned} {\widehat{\varvec{\beta }}}_{ij}^{(M_i)}(\lambda _{1}, \lambda _{2})= {\left\{ \begin{array}{ll} {\widehat{\varvec{\beta }}}_{i0j}(\lambda _{1}, \lambda _{2}), &{} j \in \text {Top}_{M_i} \\ 0 , &{} j \not \in \text {Top}_{M_i}, \end{array}\right. } \end{aligned}$$

for each fixed $\lambda _1$ and $\lambda _2$. The biomarkers with non-zero coefficients in ${\widehat{\varvec{\beta }}}_{1}={\widehat{\varvec{\beta }}}_{1}^{(M_1)}$ (resp. ${\widehat{\varvec{\beta }}}_{2}^{(M_2)}-{\widehat{\varvec{\beta }}}_{1}^{(M_1)}$) are considered as prognostic (resp. predictive) biomarkers, where the choice of $M_1$ and $M_2$ is explained in in “[Sec Sec6]” section .

To illustrate the benefits of using an additional thresholding step, we used the dataset described in “[Sec Sec4]” section. Moreover, to simplify the graphical illustrations, we also focus on the case where $\lambda _{1}=\lambda _{2}=\lambda$. Additional file 1: Figure S1 displays the number of True Positive (TP) and False Positive (FP) in prognostic and predictive biomarker identification with and without the second thresholding. We can see from this figure that the thresholding stage limits the number of false positives. Note that $\alpha _1$ and $\alpha _2$ are estimated by $\widehat{{\widetilde{\alpha }}}_{1}$ and $\widehat{{\widetilde{\alpha }}}_{2}$ defined in (10).

### Choice of the parameters $K_1, K_2$, $M_1$ and $M_2$

**Fig. 2:**
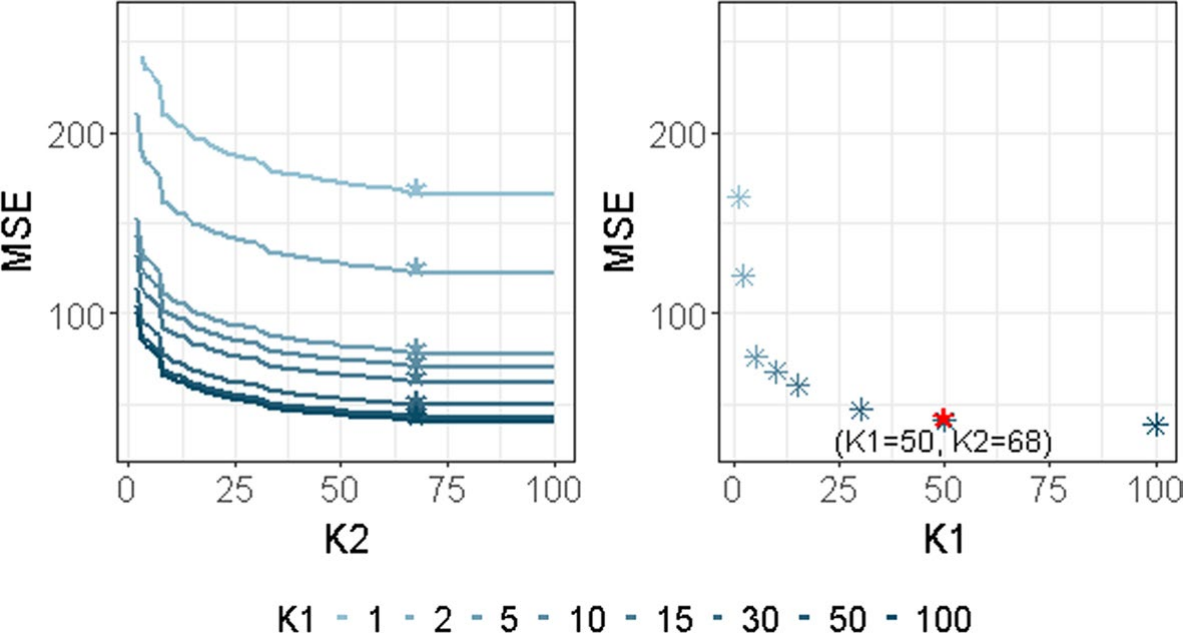
Illustration of how to choose $K_1$ and $K_2$ ($\delta =0.95$), final choice is marked with ’*’

For each $(\lambda _1, \lambda _2)$ and each $K_1$, we computed:

13 $$\begin{aligned} \widetilde{\text {MSE}}_{K_1, K_2}(\lambda _1, \lambda _2)=\Vert {\textbf{y}}-{\widetilde{{\textbf{X}}}}\widehat{{\widetilde{\varvec{\gamma }}}}^{(K1, K2)}(\lambda _1, \lambda _2)\Vert _2^2, \end{aligned}$$

where $\widehat{{\widetilde{\varvec{\gamma }}}}^{(K1, K2)}(\lambda _1, \lambda _2)=(\widehat{{\widetilde{\alpha }}}_1,\widehat{{\widetilde{\alpha }}}_2, \widehat{{\widetilde{\varvec{\beta }}}}_1^{(K_1)'},\widehat{{\widetilde{\varvec{\beta }}}}_2^{(K_2)'})$ defined in (10) and in (11). It is displayed in the left part of Fig. 2.

For each $\lambda _{1}$, $\lambda _{2}$ and a given $\delta \in (0,1)$, the parameter $\widehat{K_2}$ is then chosen as follows for each $K_1$:

$$\begin{aligned} \widehat{K_2}(\lambda _{1}, \lambda _{2})=\mathop {\textrm{Argmin}}\nolimits \left\{ K_2\ge 1 \text { s.t. }\frac{\widetilde{\text {MSE}}_{(K_1, K_2+1})(\lambda _{1}, \lambda _{2})}{\widetilde{\text {MSE}}_{(K_1,K_2)}(\lambda _{1}, \lambda _{2})} \ge \delta \right\} . \end{aligned}$$

The ${\widehat{K}}_2$ associated to each $K_1$ are displayed with ’*’ in the left part of Fig. 2. Then $\widehat{K_1}$ is chosen by using a similar criterion:

$$\begin{aligned} \widehat{K_1}(\lambda _{1}, \lambda _{2})=\mathop {\textrm{Argmin}}\nolimits \left\{ K_1\ge 1 \text { s.t. }\frac{\widetilde{\text {MSE}}_{(K_1+1, {\widehat{K}}_2})(\lambda _{1}, \lambda _{2})}{\widetilde{\text {MSE}}_{(K_1,{\widehat{K}}_2)}(\lambda _{1}, \lambda _{2})} \ge \delta \right\} . \end{aligned}$$

The values of $\widetilde{\text {MSE}}_{(K_1,{\widehat{K}}_2)}(\lambda _{1}, \lambda _{2})$ are displayed in the right part of Fig. 2 in the particular case where $\lambda _{1}=\lambda _{2}=\lambda$, $\delta =0.95$ and with the same dataset as the one used in “[Sec Sec4]” section. ${\widehat{K}}_1$ is displayed with a red star. This value of $\delta$ will be used in the following sections. However, choosing $\delta$ in the range (0.9,0.99) does not have a strong impact on the variable selection performance of our approach.

The parameters ${\widehat{M}}_1$ and ${\widehat{M}}_2$ are chosen in a similar way except that $\widetilde{\text {MSE}}_{K_1, K_2}(\lambda _1, \lambda _2)$ is replaced by $\widehat{\text {MSE}}_{M_1,M_2}(\lambda _1, \lambda _2)$ where:

$$\begin{aligned} \widehat{\text {MSE}}_{M_1,M_2}(\lambda _1, \lambda _2)=\Vert {\textbf{y}}-{\textbf{X}}{\widehat{\varvec{\gamma }}}^{(M_1,M_2)}(\lambda _1, \lambda _2)\Vert _2^2, \end{aligned}$$

with ${\widehat{\varvec{\gamma }}}^{(M_1,M_2)}(\lambda _1, \lambda _2)=(\widehat{{\widetilde{\alpha }}}_1,\widehat{{\widetilde{\alpha }}}_2, {\widehat{\varvec{\beta }}}_1^{(M_1)'},{\widehat{\varvec{\beta }}}_2^{(M_2)'})$ defined in (10) and (12). In the following, ${\widehat{\varvec{\gamma }}}(\lambda _1, \lambda _2)={\widehat{\varvec{\gamma }}}^{({\widehat{M}}_1,{\widehat{M}}_2)}(\lambda _1, \lambda _2)$.

### Estimation of $\varvec{\Sigma }_1$ and $\varvec{\Sigma }_2$

As the empirical correlation matrix is known to be a non accurate estimator of $\varvec{\Sigma }$ when *p* is larger than *n*, a new estimator has to be used. Thus, for estimating $\varvec{\Sigma }$ we adopted a cross-validation based method designed by [5] and implemented in the cvCovEst R package [6]. This method chooses the estimator having the smallest estimation error among several compared methods (sample correlation matrix, POET [11] and Tapering [7] as examples). Since the samples in treatments $t_1$ and $t_2$ are assumed to be collected from the same population, $\varvec{\Sigma }_1$ and $\varvec{\Sigma }_2$ are assumed to be equal.

### Choice of the parameters $\lambda _1$ and $\lambda _2$

**Fig. 3:**
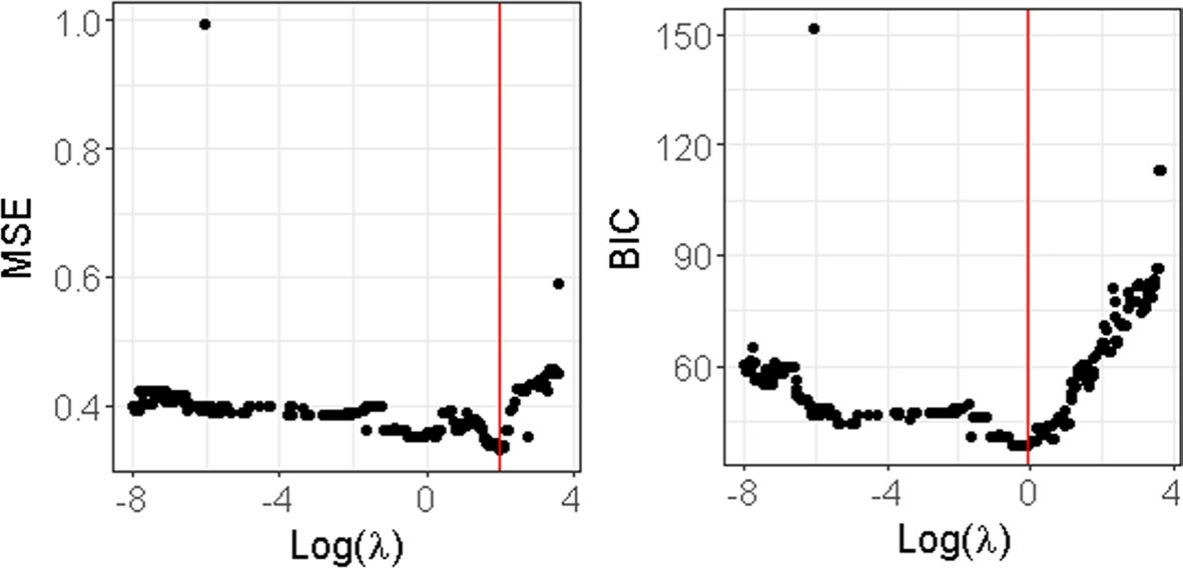
MSE and BIC for all $\lambda$. The $\lambda$ minimizing each criterion is displayed with a vertical line

For the sake of simplicity, we limit ourselves to the case where $\lambda _1=\lambda _2=\lambda$. For choosing $\lambda$ we used BIC (Bayesian information criterion) which is widely used in the variable selection field and which consists in minimizing the following criterion with respect to $\lambda$:

14 $$\begin{aligned} \text {BIC}(\lambda )= n\log (\text {MSE}(\lambda )/n)+ k(\lambda )\log (n), \end{aligned}$$

where *n* is the total number of samples, $\text {MSE}(\lambda )=\Vert {\textbf{y}}-{\textbf{X}}{\widehat{\varvec{\gamma }}}(\lambda )\Vert _2^2$ and $k(\lambda )$ is the number of non null coefficients in the OLS estimator ${\widehat{\varvec{\gamma }}}$ obtained by re-estimating only the non null components of ${\widehat{\varvec{\beta }}}_1$ and ${\widehat{\varvec{\beta }}}_2-{\widehat{\varvec{\beta }}}_1$. The values of the BIC criterion as well as those of the MSE obtained from the dataset described in “[Sec Sec4]” section are displayed in Fig. 3.

Additional file 1: Table S1 provides the True Positive Rate (TPR) and False Positive Rate (FPR) when $\lambda$ is chosen either by minimizing the MSE or the BIC criterion for this dataset. We can see from this table that both of them have TPR=1 (all true positives are identified). However, the FPR based on the BIC criterion is smaller than the one obtained by using the MSE.

Note that additional results using two different parameters $\lambda _1$ and $\lambda _2$ in the BIC criterion are provided in “[Sec Sec12]” section.

## Numerical experiments

This section presents a comprehensive numerical study by comparing the performance of our method with other regularized approaches in terms of prognostic and predictive biomarker selection. Besides the Lasso, we also compared with Elastic Net, Adaptive Lasso and WLasso [40] since they also take into account the correlations. For these compared methods, in order to directly estimate prognostic and predictive effects, ${\textbf{X}}$ and $\varvec{\gamma }$ in Model (3) were replaced by

$$\begin{aligned} {\textbf{X}}^{*}=\begin{bmatrix} {\textbf{1}}_{n_1,1} &{} {\textbf{0}}_{n_1,1} &{} {\textbf{X}}_{1} &{} {\textbf{0}}_{n_1,p} \\ {\textbf{0}}_{n_2,1} &{} {\textbf{1}}_{n_2,1} &{} {\textbf{X}}_{2} &{} {\textbf{X}}_{2} \end{bmatrix}, \end{aligned}$$

and $\varvec{\gamma }^*=(\alpha _1,\alpha _2,\varvec{\beta }_1^*,\varvec{\beta }_2^*)$, respectively, where ${\textbf{X}}_1$ and ${\textbf{X}}_2$ are defined in (1), ${\textbf{0}}_{i,j}$ (resp. ${\textbf{1}}_{i,j}$) denotes a matrix having *i* rows and *j* columns and containing only zeros (resp. ones). Note that this is the modeling proposed by [21]. The sparsity enforcing constraint was put on the coefficients $\varvec{\beta }_1^*$ and $\varvec{\beta }_2^*$ which boils down to putting a sparsity enforcing constraint on $\varvec{\beta }_1$ and $\varvec{\beta }_2-\varvec{\beta }_1$.

### Simulation setting

All simulated datasets were generated from Model (3) where the $n_1$ ($n_2$) rows of ${\textbf{X}}_{1}$ (${\textbf{X}}_{2}$) are assumed to be independent Gaussian random vectors with a covariance matrix $\varvec{\Sigma }_{1}=\varvec{\Sigma }_{2}=\varvec{\Sigma }_{bm}$, and $\varvec{\epsilon }$ is a standard Gaussian random vector independent of ${\textbf{X}}_{1}$ and ${\textbf{X}}_{2}$. We defined $\varvec{\Sigma }_{bm}$ as:

15 $$\begin{aligned} \varvec{\Sigma }_{bm}= \begin{bmatrix} \varvec{\Sigma }_{11} &{} \varvec{\Sigma }_{12} \\ \varvec{\Sigma }_{12}^{T} &{} \varvec{\Sigma }_{22} \end{bmatrix} \end{aligned}$$

where $\varvec{\Sigma }_{11}$ (resp. $\varvec{\Sigma }_{22}$) are the correlation matrix of prognostic (resp. non-prognostic) biomarkers with off-diagonal entries equal to $a_1$ (resp. $a_3$). Morever, $\varvec{\Sigma }_{12}$ is the correlation matrix between prognostic and non-prognostic variables with entries equal to $a_2$. In our simulations $(a_1,a_2, a_3)=(0.3, 0.5, 0.7)$, which is a framework proposed by [37]. We checked that the Irrepresentable Condition (IC) of [39] is violated and thus the standard Lasso cannot recover the positions of the null and non null variables. For each dataset we assumed randomized treatment allocation between standard and experimental arm with a 1:1 ratio, *i.e.* $n_1=n_2=50$. We further assume a relative treatment effect of 1 ($\alpha _{1}=0$ and $\alpha _{2}=1$). The number of biomarkers *p* varies from 200 to 2000. The number of active biomarkers was set to 10 (*i.e.* 5 purely prognostic biomarkers with $\varvec{\beta }_{1j}=\varvec{\beta }_{2j}=b_1=1$ $(j=1,...,5)$ and 5 biomarkers both prognostic and predictive with $\varvec{\beta }_{1j}=b_1$ and $\varvec{\beta }_{2j}=b_2=2$ $(j=6,...,10)$).

### Evaluation criteria

We considered several evaluation criteria to assess the performance of the methods in selecting the prognostic and predictive biomarkers: the $\text {TPR}_{\text {prog}}$ as the true positive rate (i.e. rate of active biomarkers selected) and $\text {FPR}_{\text {prog}}$ the false positive rate (i.e. rate of inactive biomarkers selected) of the selection of prognostic biomarkers, and similarly for predictive biomarkers with $\text {TPR}_{\text {pred}}$ and $\text {FPR}_{\text {pred}}$. We further note $\text {TPR}_{\text {all}}$ and $\text {FPR}_{\text {all}}$ the criterion of overall selection among all candidate biomarkers regardless their prognostic or predictive effect. The objective of the selection is to maximize the $\text {TPR}_{\text {all}}$ and minimize the $\text {FPR}_{\text {all}}$. All metrics were calculated by averaging the results of 100 replications for each scenario.

### Two parameters $\lambda _1$ and $\lambda _2$ v.s. $\lambda$ in the BIC Criterion

In this section, we compare the results obtained by choosing $\lambda _1=\lambda _2=\lambda$ as the minimizer of the BIC criterion described in (14) with those obtained by choosing the values of $\lambda _1$ and $\lambda _2$ as those minimizing the criterion (16):

16 $$\begin{aligned} \text {BIC}(\lambda _1, \lambda _2)= n\log (\text {MSE}(\lambda _1, \lambda _2)/n)+ k(\lambda _1, \lambda _2)\log (n). \end{aligned}$$

**Fig. 4:**
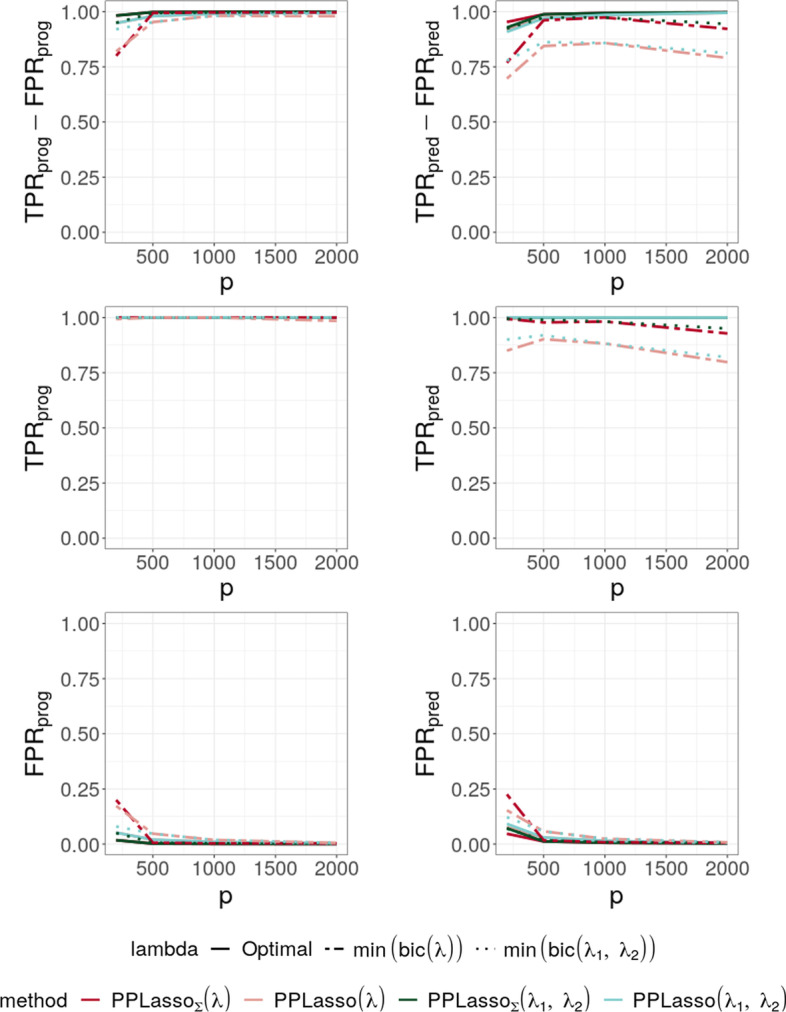
Average of (TPR-FPR) and the corresponding True Positive Rate (TPR) and False Positive Rate (FPR) for prognostic (left) and predictive (right) biomarkers. Two parameters $\lambda _1$ and $\lambda _2$ v.s. $\lambda$

Different results are presented in Fig. 4. $\text {PPLasso}_{\Sigma }$ (resp. $\text {PPLasso}$) corresponds to the results of the method by using the true (resp. estimated) matrix $\varvec{\Sigma }_{bm}$. For estimating $\varvec{\Sigma }_{bm}$, we used the approach explained in “[Sec Sec7]” section . Different choices of parameters are also given: “optimal”, “$\text {min(bic}(\lambda ))$” and “$\text {min(bic}(\lambda _1, \lambda _2))$”. The first one uses as a value of the parameters the one maximizing $(\text {TPR}_{\text {all}}-\text {FPR}_{\text {all}})$, the second one uses the approach presented in (14) and the last one uses the approach described in (16).

We observed that the results with two tuning parameters $(\lambda _1, \lambda _2)$ were slightly better than those with a single parameter $\lambda$. However, the gap is very small and almost invisible when *p* increases. For this reason, we limited ourselves to a single tuning parameter $\lambda$ in the following.

### Biomarker selection results

In order to compare the performance of our approach to the best performance that could be reached by Elastic Net, Lasso, Adaptive Lasso and WLasso, we used for these methods the “optimal” parameters namely those maximizing $(\text {TPR}_{\text {all}}-\text {FPR}_{\text {all}})$. The first three methods were implemented with the glmnet R package, the best parameter $\alpha$ involved in Elastic Net was chosen in the set $\{0.1, 0.2,\dots ,0.9\}$. WLasso was implemented with the WLasso R package. The choice of “min(bic)” is only applied to our method and corresponds to a choice of $\lambda$ that could be used in practical situations. For ease of presentation, the abbreviation EN (resp. AdLasso) refers to Elastic Net (resp. Adaptive Lasso) in the following.

**Fig. 5:**
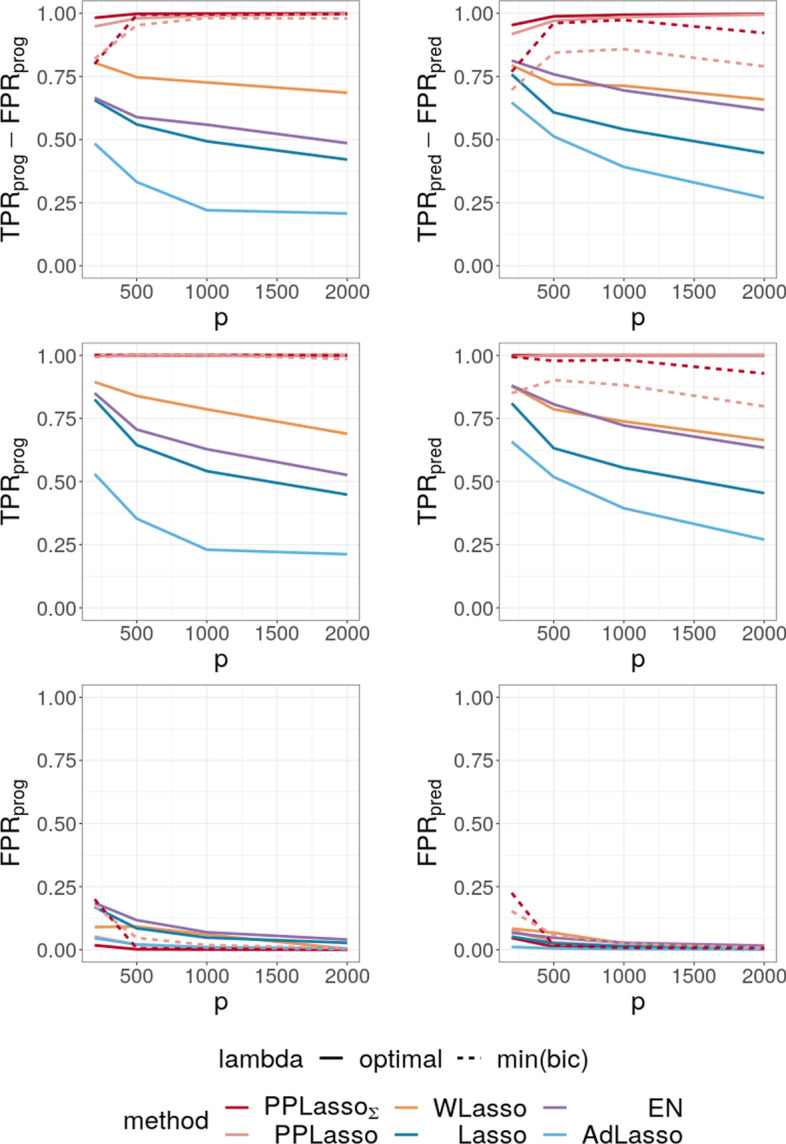
Average of (TPR-FPR) and the corresponding True Positive Rate (TPR) and False Positive Rate (FPR) for prognostic (left) and predictive (right) biomarkers

Figure 5 shows the selection performance of PPLasso and other compared methods in the simulation scenario presented in “Simulation setting” section. PPLasso achieved to select all prognostic biomarkers ($\text {TPR}_{\text {prog}}$ almost 1) even for large *p*, with limited false positive prognostic biomarkers selected. As compared to the optimal $\lambda$ maximizing $(\text {TPR}_{\text {all}}-\text {FPR}_{\text {all}})$, the one selected with the BIC tends to select some false positives (average: 33 ($\text {FPR}_{\text {prog}} = 0.17$) for $p=200$ and 10 ($\text {FPR}_{\text {prog}} = 0.005$) for $p=2000$). The results obtained from the oracle and estimated $\varvec{\Sigma }_{bm}$ are comparable. Selection performance of predictive biomarkers is slightly lowered as compared to prognostic biomarkers. Even if the false positive selection is quite similar between prognostic and predictive biomarkers, PPLasso missed some true predictive biomarkers when $\lambda$ is selected with the BIC criterion (average $\text {TPR}_{\text {pred}} =$ 0.98 and 0.80 for oracle and estimated $\varvec{\Sigma }_{bm}$, respectively, with $p=2000$). In this scenario where the IC is violated, PPLasso globally outperforms Lasso, Elastic Net, Adaptive Lasso and WLasso. Thanks to the whitening technique used in WLasso, it achieved higher selection accuracy than the other three methods. Although Elastic Net showed higher TPR than Lasso and Adaptive Lasso, they all failed in selecting all truly prognostic and predictive biomarkers, and the number of missed active biomarkers increased with the dimension *p*. For example, for Elastic Net, $\text {TPR}_{\text {prog}}$ = 0.85 and 0.53, $\text {TPR}_{\text {pred}}$ = 0.81 and 0.61 for $p = 200$ and 2000, respectively.

#### Impact of the correlation matrix $\varvec{\Sigma }$

To evaluate the impact of the correlation matrix on the selection performance of the methods, additional scenarios are presented where the IC is satisfied:

1. Compound symmetry structure where all biomarkers are equally correlated with a correlation $\rho =0.5$;
2. Independent setting where $\varvec{\Sigma }_{bm}$ is the identity matrix.

**Fig. 6:**
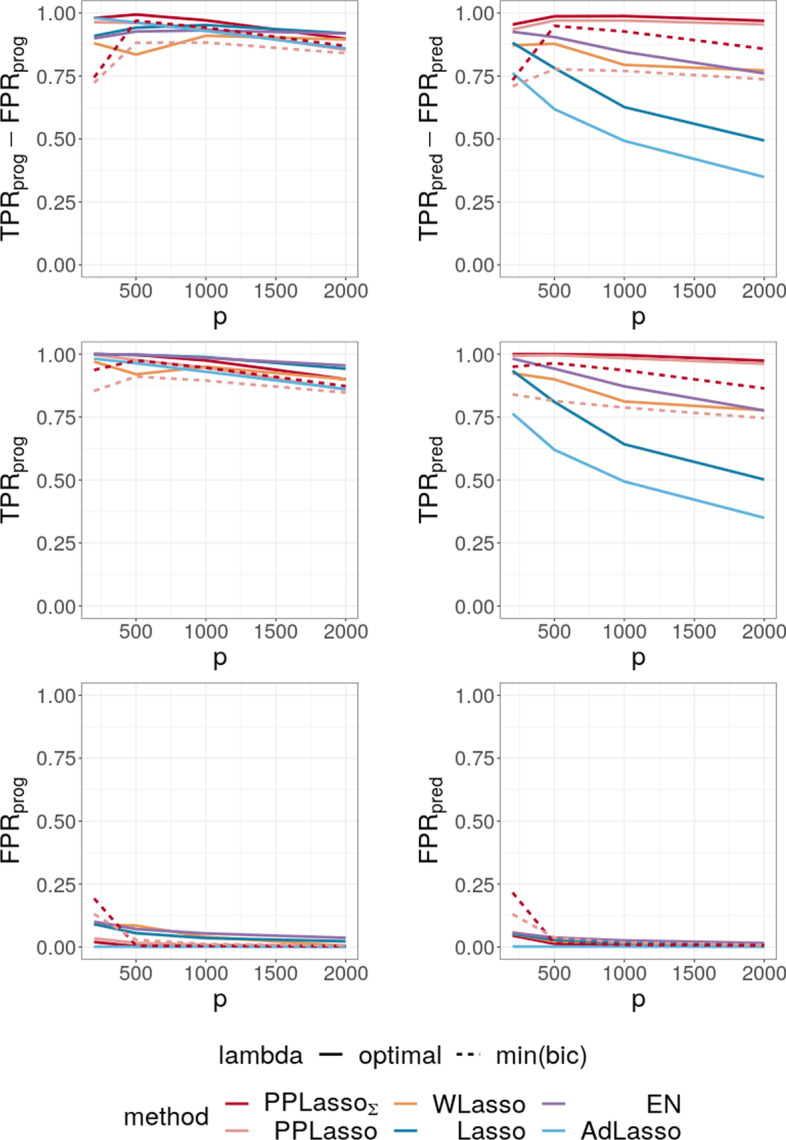
Average of (TPR-FPR) and the corresponding True Positive Rate (TPR) and False Positive Rate (FPR) for prognostic (left) and predictive (right) biomarkers for the compound symmetry correlation structure

For the scenario with compound symmetry structure displayed in Fig. 6, all the methods successfully identified the true prognostic biomarkers ($\text {TPR}_{\text {prog}}$ close to 1 even for large *p*) with limited false positive selection. On the other hand, the compared methods (Lasso, ELastic Net, Adaptive Lasso and WLasso) missed some predictive biomarkers especially when *p* increases.

On the contrary, PPLasso successfully identified almost all predictive biomarkers with the optimal choice of $\lambda$. Moreover, even when $\lambda$ is selected by minimizing the BIC criterion (min(bic)), $\text {PPLasso}_{\text {est}}$ outperformed Lasso and Adaptive Lasso when $p > 500$ with relatively stable $\text {TPR}_{\text {pred}}$ and $\text {FPR}_\text {pred}$ as *p* increases.

**Fig. 7:**
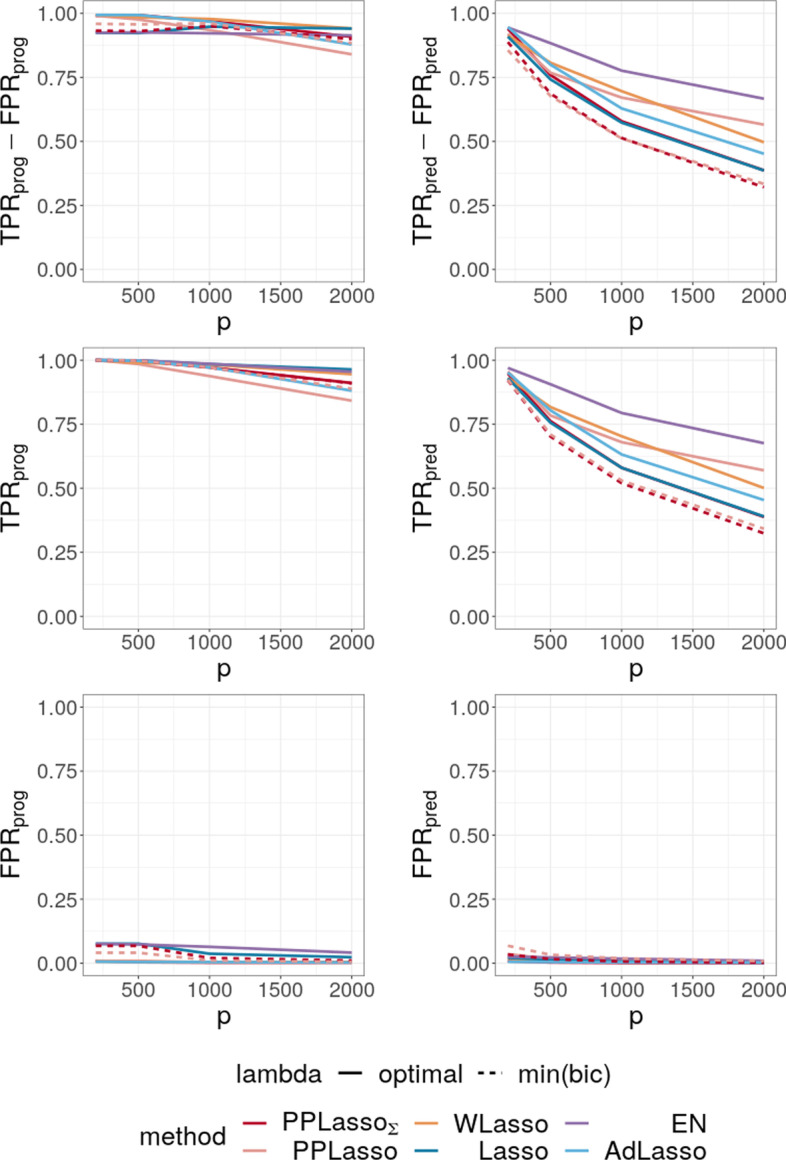
Average of (TPR-FPR) and the corresponding True Positive Rate (TPR) and False Positive Rate (FPR) for prognostic (left) and predictive (right) biomarkers (independent setting)

For the independent setting, as displayed in Fig. 7, prognostic biomarkers were globally well identified by all the compared methods with a slightly higher $\text {TPR}_{\text {prog}}$ for Lasso and ELastic Net as compared to PPLasso but also with a slightly higher $\text {FPR}_{\text {prog}}$. With regards to predictive biomarkers, PPLasso using $\varvec{\Sigma }_{bm}$ (oracle) performed also similarly to the Lasso, which is reasonable since no transformation has been used in PPLasso. On the other hand, even if PPLasso with $\lambda$ selected with “min(bic)” performed similarly with PPLasso with optimal $\lambda$ for relatively small *p*, the selection performance is altered for large *p* and even if the performance is higher than Lasso and Adaptive Lasso, it is smaller than the one of Elastic Net.

#### Impact of the effect size of active biomarkers

To evaluate the impact of the effect size on biomarker selection performance, the scenario presented in “Simulation setting” section  was considered with different values of $b_{2}$: 1.5, 2 and 2.5.

Since the effect size of prognostic biomarkers did not change, the comparison focused on predictive biomarkers. As expected, the reduction of the effect size makes the biomarker selection harder, especially for Lasso, Elastic Net and Adaptive Lasso where the predictive biomarker selection is limited when $b_{2}=1.5$: for Lasso when $p=2000$, $\text {TPR}_{\text {pred}}$ = 0.45 (resp. 0.22) for $b_{2}=2$ (resp. 1.5), see Fig. 5 and Additional file 1: Figure S2. The selection performance of PPLasso when $\lambda$ is selected with min(bic) is also reduced by decreasing $b_2$, especially when $\varvec{\Sigma }_{bm}$ is also estimated. Nevertheless, the selection performance of PPLasso remains better than for most of the other compared methods for which the performance displayed are associated to the optimal value of $\lambda$. Surprisingly, WLasso performed better than PPLasso with estimated $\lambda$ in this scenario. On the other hand, even with limited effect size, PPLasso with optimal $\lambda$ identified all predictive biomarkers with very limited false positive selection. When $b_2$ was increased to 2.5, the selection performance for all methods is improved and the results for PPLasso with estimated $\lambda$ was close to the ones with the optimal $\lambda$ as displayed in Additional file 1: Figure S3. As compared with PPLasso, for which the selection performance remained stable as *p* increased, Lasso, Elastic Net, Adaptive Lasso and WLasso were more impacted by the value of *p* since the true positive selection decreased as *p* increased. As an example, for the Lasso, $\text {TPR}_{\text {pred}}$ =0.95 (resp. 0.65) for $p = 200$ (resp. 2000).

#### Impact of the number of predictive biomarkers

The impact of the number of true predictive biomarkers was assessed by increasing the number of predictive biomarkers from 5 to 10 in the scenario presented in “Simulation setting” section. When the number of predictive biomarkers increased, the impact on PPLasso is almost negligible, especially for prognostic biomarker identification. However, for the other methods, we can see from Additional file 1: Figure S4 that it became even harder to identify predictive biomarkers. The impact on WLasso was less obvious, while for the other methods, $\text {TPR}_{\text {pred}}$ decreased compared to Fig. 5, especially for large *p* (e.g. $\text {TPR}_{\text {pred}} =$ 0.12, 0.18, and 0.02 for Lasso, Elastic Net and Adaptive Lasso respectively when $p = 2000$).

#### Impact of the dimension of the dataset

In this section, we studied a different sample size: *n* = 50 with $n_1=n_2=25$ and a different number of biomarkers: *p* = 5000.

We can see from Additional file 1: Figure S5 that for $p=5000$, the selection performance of PPLasso is not altered as compared with $p=2000$ while the compared methods have more difficulties to identify both prognostic and predictive biomarkers.

When the sample size is smaller (*n* = 50), we can see from Additional file 1: Figure S6 that the ability to identify prognostic and predictive biomarkers decreased for all the methods. However, PPLasso still outperformed the others with higher $\text {TPR}_{\text {prog}}$ and $\text {TPR}_{\text {pred}}$ and lower $\text {FPR}_{\text {prog}}$ and $\text {FPR}_{\text {pred}}$.

## Application to transcriptomic and proteomic data

### Application to the RV144 clinical trial transcriptomic data

We applied the previously described methods to publicly available transcriptomic data from the RV144 vaccine trial [24]. This trial showed reduced risk of HIV-1 acquisition by 31.2% with vaccination with ALVAC and AIDSVAX as compared to placebo. Transcriptomic profiles of in vitro HIV-1 Env-stimulated peripheral blood mononuclear cells (PBMCs) obtained pre-immunization and 15 days after the immunization (D15) from both 40 vaccinees and 10 placebo recipients were generated to better understand underlying biological mechanisms.

For illustration purpose, the absolute change at D15 in gene mTOR was considered as the continuous endpoint (response). mTOR plays a key role in mTORC1 signaling pathway which has been shown to be associated with risk of HIV-1 acquisition [14, 1]. The gene expression has been normalized as in the original publication of [14]. After removing non-annotated genes (LOCxxxx and HS.xxxx), the top 2000 genes with the highest empirical variances were included as candidate biomarkers for prognostic and predictive identification from PPLasso and the compared methods. The penalty parameter $\lambda$ for the Lasso and Adaptive Lasso, the parameters $\lambda$ and $\alpha$ for Elastic Net were selected through the classical cross-validation approach. For PPLasso, $\lambda$ was selected based on the criterion described in “[Sec Sec8]” section .

**Fig. 8:**
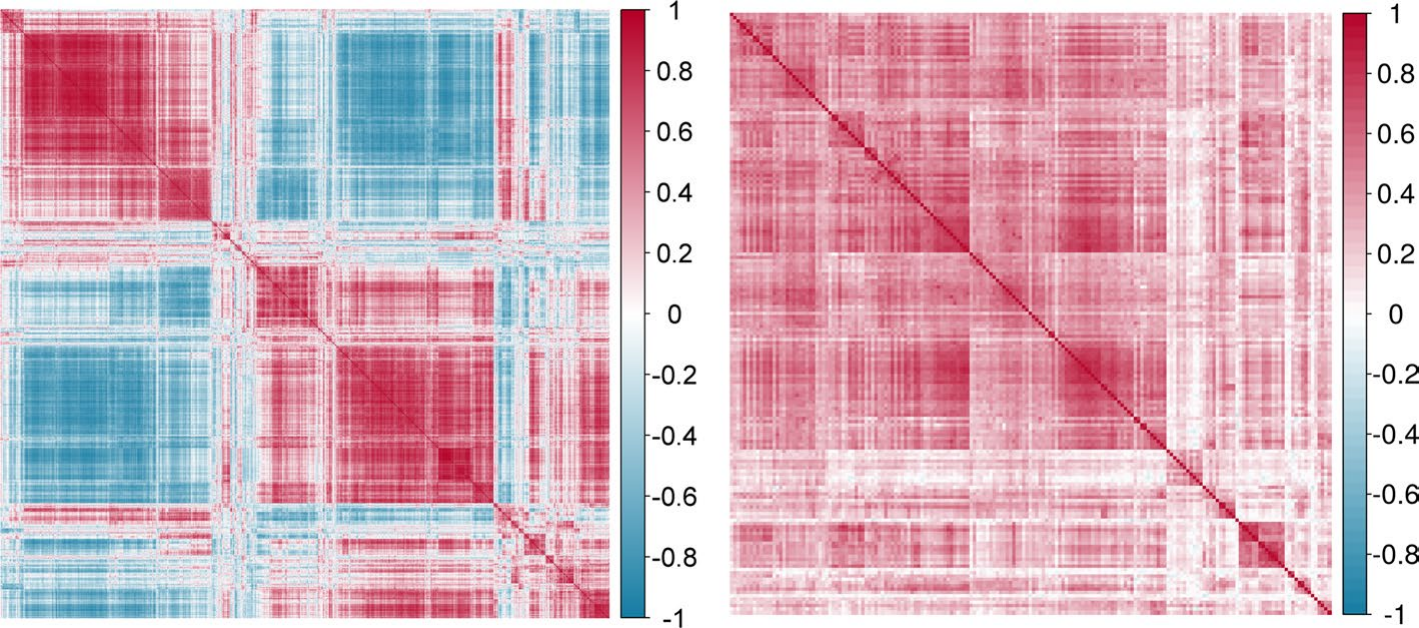
Heatmaps of the correlation matrices estimated by the cvCovEst R package. Left: transcriptomic data from clinical trial RV144. Right: proteomic data from clinical trial NCT01241591

The estimation of $\varvec{\Sigma }$ was obtained by comparing several candidate estimators from the cvCovEst R package and by selecting the estimator having the smallest estimation error. In this application, the combination of the sample covariance matrix and a dense target matrix (*denseLinearShrinkEst*) derived by [18] provides the smallest estimation error. Figure 8 (left) displays the estimated $\varvec{\Sigma }$ and highlights the strong correlation between the genes. Additional file 1: Table S2 gives details on the compared estimators.

Prognostic and predictive genes selected by PPLasso, Lasso, Elastic Net and Adaptive Lasso are listed in Table 1. The number of genes selected are similar for all the compared methods, except for a slightly higher number of predictive genes selected by PPLasso. Lasso, Elastic Net and Adaptive Lasso selected very similar sets of prognostic and predictive genes. The intersection between PPLasso and others is moderate (2 prognostic genes (SLAMF7 and TNFRSF6B), 3 predictive genes (YTHDC1, MS4A7 and RPL21)).

To have a better overview of the prognostic and predictive genes selected by the different methods and their associated roles, pathway analysis was carried out via the REACTOME tool (https://reactome.org/), where over-representation analysis (ORA) was performed. ORA is used to determine if a set of genes shares more genes with a pathway than we would expect by chance, evaluated by a *p*-value. Table S3 (prognostic biomarkers) and Additional file 1: Table S4 (predictive biomarkers) showed the identified pathways with a *p*-value smaller than 0.01. For prognostic biomarkers, there was no pathway identified by WLasso. Most of the pathways identified by Adaptive Lasso were also identified by Elastic Net. Lasso identified a large number of pathways, but some of them may not be related to HIV. PPLasso identified three pathways (also identified by Elastic Net and Lasso). Interestingly, TNFR2 non-canonical NF-kB pathway that was already identified by Fourati et al. [14], is associated with the risk of HIV acquisition in the placebo group; the implication of regulatory T-cells on HIV-1 has also been widely discussed in the literature (e.g., [17]). For predictive biomarkers identified by different methods, Lasso, Elastic Net, and Adaptive Lasso identified comparable pathways, while PPLasso and WLasso share similar ones and different from the other methods. Among the pathways identified by PPLasso, NOD1/2 Signaling Pathway and Toll-like Receptor Cascades pathway are reported as potential targeted adjuvants for HIV-1 vaccines [22]. In addition, RIPK1-mediated regulated necrosis pathway has also been investigated as targets for HIV-1 protease activity during infection [33].

**Table 1:** Selected genes from PPLasso, Lasso, Elastic Net, Adaptive Lasso and WLasso

	Prognostic genes	Predictive genes
PPLasso	HAPLN3, **SLAMF7**, GTF3C5, FAM46A, SH3PXD2B, TM4SF1, **TNFRSF6B**, TNFRSF18, TRPM2	TLR8, **YTHDC1**, NUCKS1, **BIRC3**, SLAMF7, NFATC2IP, BOK, MGRN1, KIAA0492, SLC25A36, HMGN2, P2RY5, **RPL21**, **MS4A7**, RPL12P6
Lasso	DKFZp434K191, NUCKS1, MAFF, **SLAMF7**, HIST2H2AC, HIST1H4C, IL8, **TNFRSF6B**, TNFRSF18, SCAND1	DKFZp434K191, **YTHDC1**, VMO1, BOLA2, HIST1H4C, **RPL21**, **MS4A7**
Elastic Net	DKFZp434K191, NUCKS1,SNURF, MAFF, **SLAMF7**, IL8, ZBP1, **TNFRSF6B**, ZAK, TNFRSF18, SCAND1, NME1-NME2, DNM1L, RNF146, NPEPL1	DKFZp434K191, **YTHDC1**, PMP22, VMO1, BOLA2, HIST1H4C, **RPL21**, **MS4A7**,RAB11FIP1
Adaptive Lasso	NUCKS1,SNURF, MAFF, **SLAMF7**, IL8, ZBP1, **TNFRSF6B**, NME1-NME2, DNM1L, RNF146	**YTHDC1**, PMP22, VMO1, BOLA2, HIST1H4C, **MS4A7**, **RPL21**
WLasso	**SLAMF7**, EEF1A1P22, RPL21P87, LRRN3, MYOM2 RPS2P5, NME1-NME2, DNM1L, RNF14	**YTHDC1**, SCARA2, KSP37, **BIRC3**

Commonly selected genes are in bold

### Application to the NCT01241591 clinical trial proteomic data

Baseline blood samples of 173 samples ($n = 81$ and 92) were taken from patients included in a randomized phase 3 clinical trial comparing the efficacy and safety of tofacitinib and etanercept in moderate-to-severe chronic plaque psoriasis (https://clinicaltrials.gov/ct2/show/NCT01241591) [2]. From these samples, 92 inflammation-associated proteins and 65 cardiovascular disease-associated proteins were measured. Response to treatment was evaluated on the change in PASI score from baseline to week 12. The aim of this application is to identify potential prognostic (proteins associated with the clinical endpoint under standard therapy: etanercept) and predictive biomarkers (proteins differentially associated with the clinical endpoint between etanercept and tofacitinib, aiming to identify patients more likely to benefit from a specific treatment). Figure 8 (right) displays the estimated $\varvec{\Sigma }$ and shows positive correlations between the proteins after standardization. Prognostic and predictive proteins selected by different methods are listed in Table 2. Among the identified prognostic proteins, IL-8 (identified by PPLasso and WLasso) and IL-17C (identified by Elastic Net and WLasso) both contribute to the IL-17 pathway of psoriasis pathogenesis mechanism [4]. For predictive proteins, CSF-1, identified by PPLasso, has been shown to be in the core signatures to predict tofacitinib treatment response developed by Tomalin et al. [32]. Lasso selected no proteins. Adaptive Lasso selected only one predictive protein.

**Table 2:** Selected proteins from PPLasso, Lasso, Elastic Net, Adaptive Lasso and WLasso

	Prognostic	Predictive
PPLasso	**IL-8**, SLAMF1, **IL-24**, TIE2, NT-pro-BNP	CCL19, CD40-L, CSF-1
Lasso	–	–
Elastic Net	**IL-17C**, SCF, IL-18, IL-18R1, IL-12B, **IL-24**, CCL28, DNER, CD40-L, hK11, MMP-3, mAmP, LEP	AM, IL27-A
Adaptive Lasso	–	ECP
WLasso	**IL-8**, **IL-17C**, MCP-1, SIRT2, CCL20	ARTN, IL-20, REN, AGRP

All models are adjusted on the baseline PASI score

Proteins commonly identified by at least two methods are in bold

## Conclusion

We propose a new method named PPLasso to simultaneously identify prognostic and predictive biomarkers. PPLasso is particularly interesting for dealing with high dimensional omics data when the biomarkers are highly correlated, which is a framework that has not been thoroughly investigated yet. From various numerical studies with or whithout strong correlation between biomarkers, we highlighted the strength of PPLasso in well identifying both prognostic and predictive biomarkers with limited false positive selection. The current method is only dedicated to the analysis of continuous responses through ANCOVA type models. However, it will be the subject of a future work to extend it to other challenging contexts, such as classification or survival analysis.

## Supplementary Information

**Additional file 1. **Supplementary material.

## Data Availability

The transcriptomic dataset is available in the Gene Expression Omnibus (GEO) database, with number GSE103671 (https://www.ncbi.nlm.nih.gov/geo/query/acc.cgi?acc=GSE103671). The proteomic dataset is available in the Gene Expression Omnibus (GEO) database, with number GSE136435 (https://www.ncbi.nlm.nih.gov/geo/query/acc.cgi?acc=GSE136435). PPLasso is an R package that is freely available on the Comprehensive R Archive Network (https://cran.r-project.org/src/contrib/Archive/PPLasso/), with vignette included.

## Abbreviations

Lasso: Least absolute shrinkage and selection operator
PPLasso: Prognostic Predictive Lasso
BIC: Bayesian information criterion
MSE: Mean squared error
TPR: True positive rate
FPR: False positive rate
EN: Elastic net
ANCOVA: Analysis of covariance
PASI: Psoriasis area and severity Index

## References

[1] Akbay B, Shmakova A, Vassetzky Y, Dokudovskaya S (2020). Modulation of mTORC1 signaling pathway by HIV-1. Cells.

[2] Bachelez H, Peter CM, Robert S, Alexey K, Fernando V, Joo-Heung L, Vladimir Y (2015). Tofacitinib versus etanercept or placebo in moderate-to-severe chronic plaque psoriasis: a phase 3 randomised non-inferiority trial. Lancet.

[3] Ballman KV (2015). Biomarker: predictive or prognostic?. J Clin Oncol.

[4] Blauvelt A, Chiricozzi A (2018). The immunologic role of IL-17 in psoriasis and psoriatic arthritis pathogenesis. Clin Rev Allergy Immunol.

[5] Boileau P, Hejazi NS, van der Laan MJ, Dudoit S. Cross-validated loss-based covariance matrix estimator selection in high dimensions. 2021. arXiv preprint arXiv:2102.09715. 10.1080/10618600.2022.2110883 PMC10237052 37273839

[6] Boileau P, Hejazi NS, van der Laan MJ, Dudoit S (2021). cvCovEst: cross-validated covariance matrix estimator selection and evaluation in R. J Open Source Softw.

[7] Cai T, Zhang C-H, Zhou H (2010). Optimal rates of convergence for covariance matrix estimation. Ann Stat.

[8] Clark G (2008). Prognostic factors versus predictive factors: examples from a clinical trial of erlotinib. Mol Oncol.

[9] Fan J, Li R. Statistical challenges with high dimensionality: feature selection in knowledge discovery. In: Proc. Madrid Int. Congress of Mathematicians; 2006. p. 3.

[10] Fan J, Lv J (2009). A selective overview of variable selection in high dimensional feature space. Stat Sin.

[11] Fan J, Liao Y, Mincheva M (2013). Large covariance estimation by thresholding principal orthogonal complements. J R Stat Soc Ser B Stat Methodol.

[12] Faraway JJ. Practical regression and ANOVA using R. University of Bath. 2002.

[13] Foster J, Taylor J, Ruberg S (2011). Subgroup identification from randomized clinical trial data. Stat Med.

[14] Fourati S, Ribeiro S, Blasco Lopes F, Talla A, Lefebvre F, Cameron M, Kaewkungwal J, Pitisuttithum P, Nitayaphan S, Rerks-Ngarm S, Kim J, Thomas R, Gilbert P, Tomaras G, Koup R, Michael N, McElrath M, Gottardo R, Sékaly R (2019). Integrated systems approach defines the antiviral pathways conferring protection by the RV144 HIV vaccine. Nat Commun.

[15] Giannos P, Kechagias K, Gal A (2021). Identification of prognostic gene biomarkers in non-small cell lung cancer progression by integrated bioinformatics analysis. Biology.

[16] He Y, Luo Y, Huang L, Zhang D, Wang X, Ji J, Liang S (2021). New frontiers against sorafenib resistance in renal cell carcinoma: from molecular mechanisms to predictive biomarkers. Pharmacol Res.

[17] Kleinman AJ, Sivanandham R, Pandrea I, Chougnet CA, Apetrei C (2018). Regulatory T cells as potential targets for HIV cure research. Front. Immunol..

[18] Ledoit O, Wolf M (2020). The power of (non-)linear shrinking: a review and guide to covariance matrix estimation. J Financ Econom..

[19] Lipkovich I, Dmitrienko A (2014). Strategies for identifying predictive biomarkers and subgroups with enhanced treatment effect in clinical trials using sides. J Biopharm Stat.

[20] Lipkovich I, Dmitrienko A, Denne J, Enas G (2011). Subgroup identification based on differential effect search (sides)—a recursive partitioning method for establishing response to treatment in patient subpopulations. Stat Med.

[21] Lipkovich I, Dmitrienko A, D’Agostino Sr RB (2017). Tutorial in biostatistics: data-driven subgroup identification and analysis in clinical trials. Stat Med.

[22] Liu J, Ostrowski M (2017). Development of targeted adjuvants for HIV-1 vaccines. AIDS Res Ther.

[23] McDonald J (2009). Handbook of biological statistics.

[24] Rerks-Ngarm S, Pitisuttithum P, Nitayaphan S, Kaewkungwal J, Chiu J, Paris R, Premsri N, Namwat C, De Souza M, Benenson M, Gurunathan S, Tartaglia J, McNeil J, Francis D, Stablein D, Birx D, Chunsuttiwat S, Khamboonruang C, Kim J (2009). Vaccination with ALVAC and AIDSVAX to prevent HIV-1 infection in Thailand. N Engl J Med.

[25] Saeys Y, Inza I, Larranaga P (2007). A review of feature selection techniques in bioinformatics. Bioinformatics.

[26] Sechidis K, Papangelou K, Metcalfe PD, Svensson D, Weatherall J, Brown G (2018). Distinguishing prognostic and predictive biomarkers: an information theoretic approach. Bioinformatics.

[27] Smith G (2018). Step away from stepwise. J Big Data.

[28] Ternès N, Rotolo F, Heinze G, Michiels S (2016). Identification of biomarker-by-treatment interactions in randomized clinical trials with survival outcomes and high-dimensional spaces. Biom J.

[29] Tian L, Alizadeh A, Gentles A, Tibshirani R (2012). A simple method for estimating interactions between a treatment and a large number of covariates. J Am Stat Assoc.

[30] Tibshirani R (1996). Regression shrinkage and selection via the lasso. J R Stat Soc Ser B (Stat Methodol).

[31] Tibshirani RJ, Taylor J (2011). The solution path of the generalized lasso. Ann Stat.

[32] Tomalin L, Kim J, Correa da Rosa J, Lee J, Fitz L, Berstein G, Valdez H, Wolk R, Krueger J, Suárez-Fariñas M (2020). Early quantification of systemic inflammatory proteins predicts long-term treatment response to tofacitinib and etanercept. J Investig Dermatol.

[33] Wagner RN, Reed JC, Chanda SK (2015). HIV-1 protease cleaves the serine-threonine kinases RIPK1 and RIPK2. Retrovirology.

[34] Wang H, Lengerich B, Aragam B, Xing E (2019). Precision lasso: accounting for correlations and linear dependencies in high-dimensional genomic data. Bioinformatics.

[35] Wang X, Leng C (2016). High dimensional ordinary least squares projection for screening variables. J R Stat.

[36] Windeler J (2000). Prognosis—what does the clinician associate with this notion?. Stat Med.

[37] Xue F, Qu A. Variable selection for highly correlated predictors. 2017. arXiv preprint arXiv:1709.04840.

[38] Zhao N, Guo M, Wang K, Zhang C, Liu X (2020). Identification of pan-cancer prognostic biomarkers through integration of multi-omics data. Front Bioeng Biotechnol.

[39] Zhao P, Yu B (2006). On model selection consistency of lasso. J Mach Learn Res.

[40] Zhu W, Lévy-Leduc C, Ternès N (2021). A variable selection approach for highly correlated predictors in high-dimensional genomic data. Bioinformatics.

[41] Zou H, Hastie T (2005). Regularization and variable selection via the elastic net. J R Stat Soc Ser B (Statistical Methodology).

[42] Zou H (2006). The adaptive lasso and its oracle properties. J Am Stat Assoc.

