# Supplementary material for: Identification of prognostic and predictive biomarkers in high-dimensional data with PPLasso
Source: BMC Bioinformatics. 2023 Jan 23;24:25. doi: 10.1186/s12859-023-05143-0 (PMC9869528; doi:10.1186/s12859-023-05143-0)
Supplement: Supplementary file 1 — Additional file 1. Supplementary material. [file 12859_2023_5143_MOESM1_ESM.pdf]

SUPPLEMENTARY MATERIAL

Abstract. This supplementary material provides additional numerical experiments, figures and a table for the paper: "Identification of prognostic and predictive biomarkers in high-dimensional data with PPLasso".

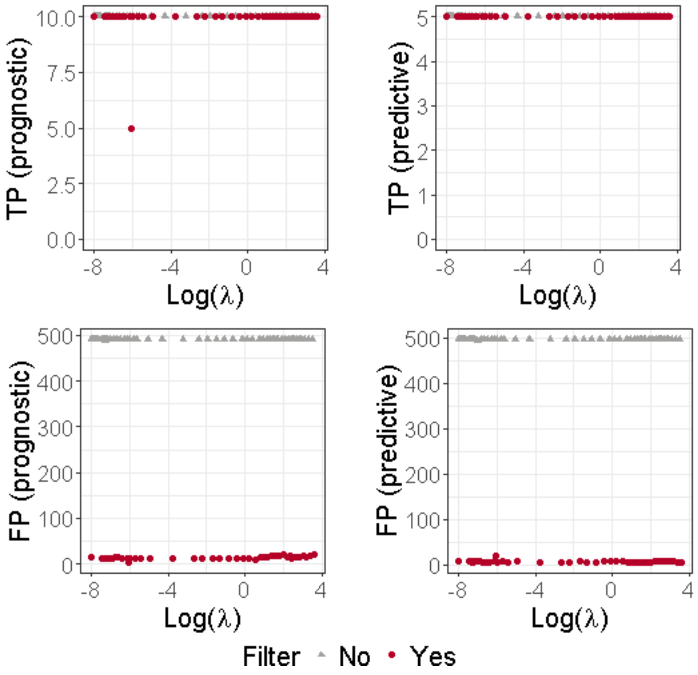

Figure S1. Number of True Positives and True Negatives for $\widehat{\boldsymbol{\beta}}$ and $\widehat{\boldsymbol{\beta}}_0$ on prognostic/predictive biomarkers.

	MSE	BIC
TPR(prognostic)	1.000	1.000
FPR(prognostic)	0.038	0.024
TPR(predictive)	1.000	1.000
FPR(predctive)	0.008	0.006

Table S1. TPR and FPR associated to prognostic and predictive biomarker identification with the λ chosen in Figure 3.

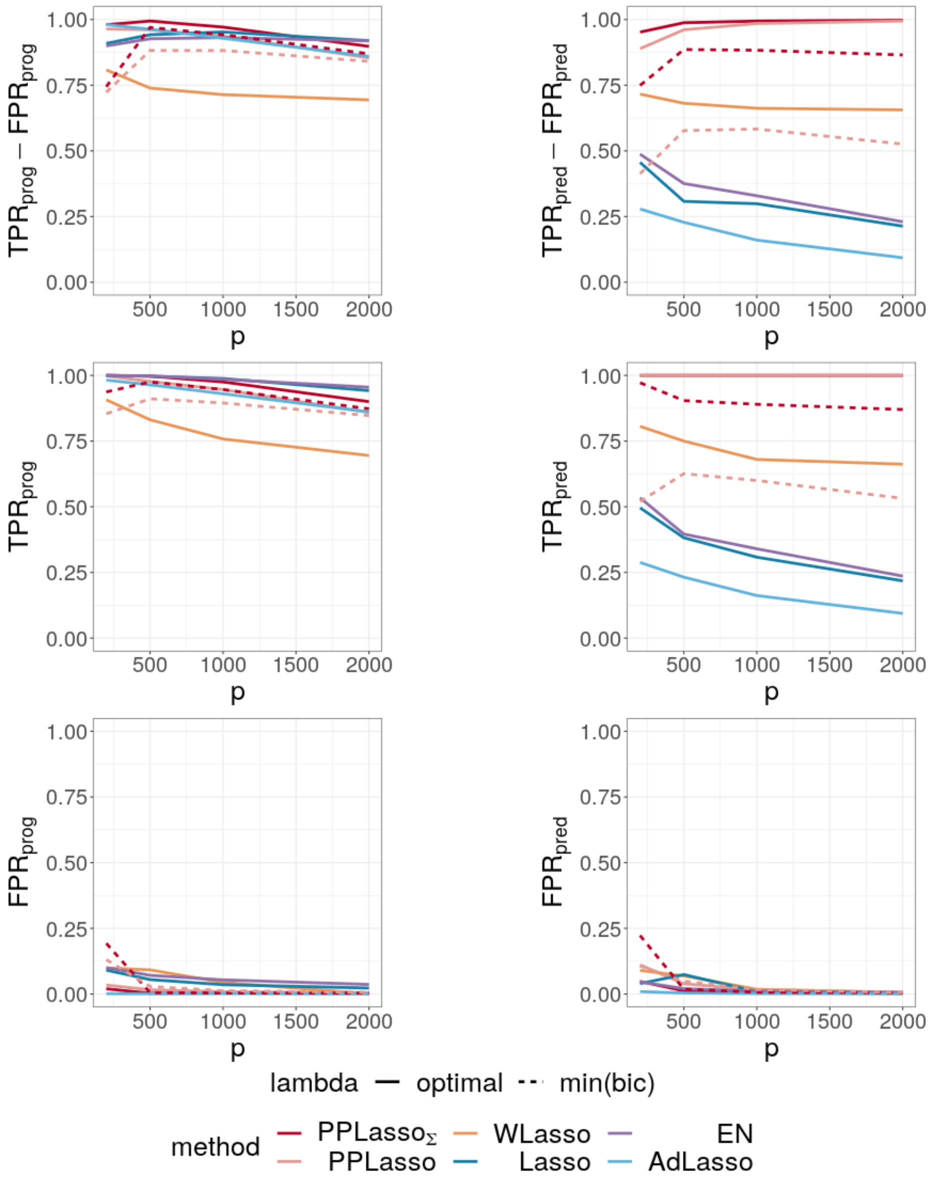

FIGURE S2. Average of (TPR-FPR) and the corresponding True Positive Rate (TPR) and False Positive Rate (FPR) for prognostic (left) and predictive (right) biomarkers ($b_2 = 1.5$).

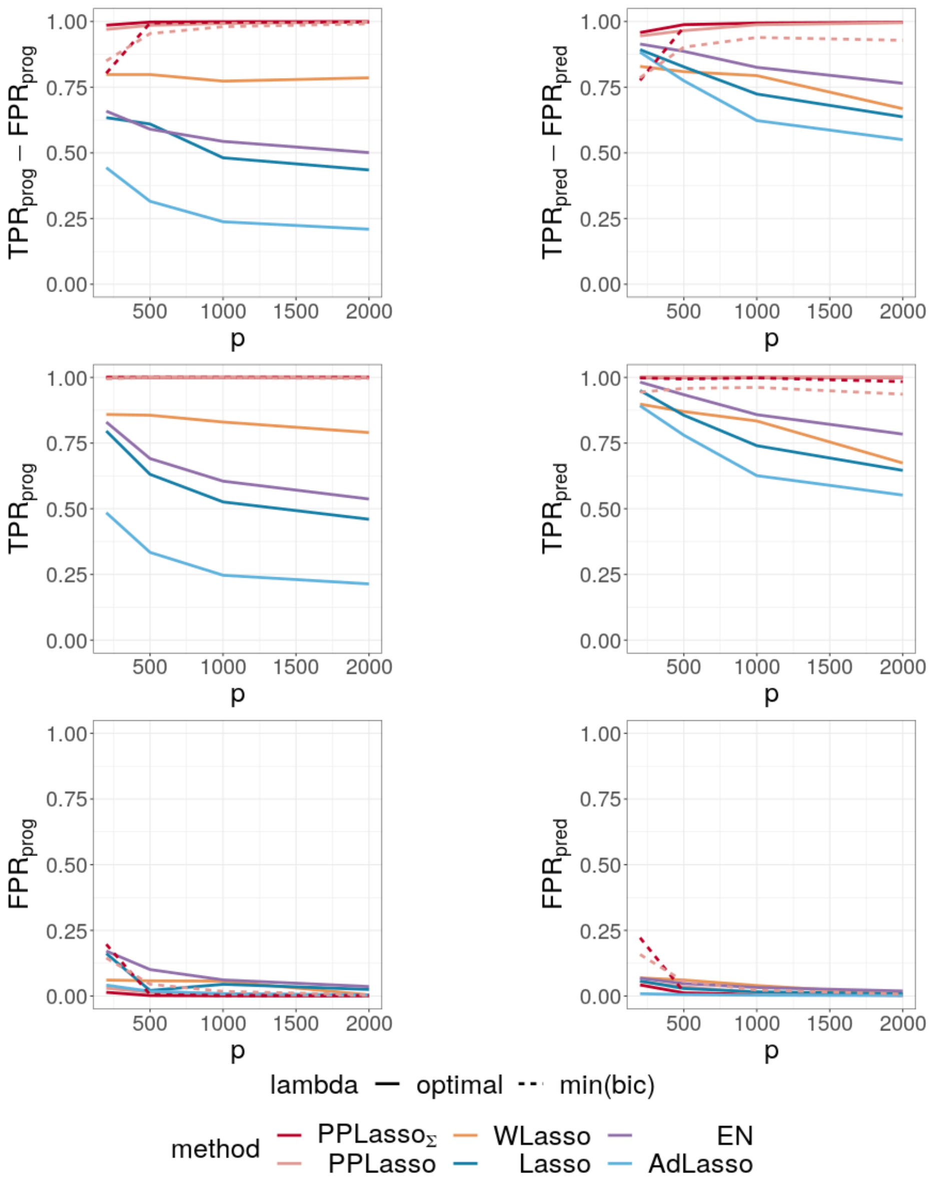

FIGURE S3. Average of (TPR-FPR) and the corresponding True Positive Rate (TPR) and False Positive Rate (FPR) for prognostic (left) and predictive (right) biomarkers ($b_2 = 2.5$).

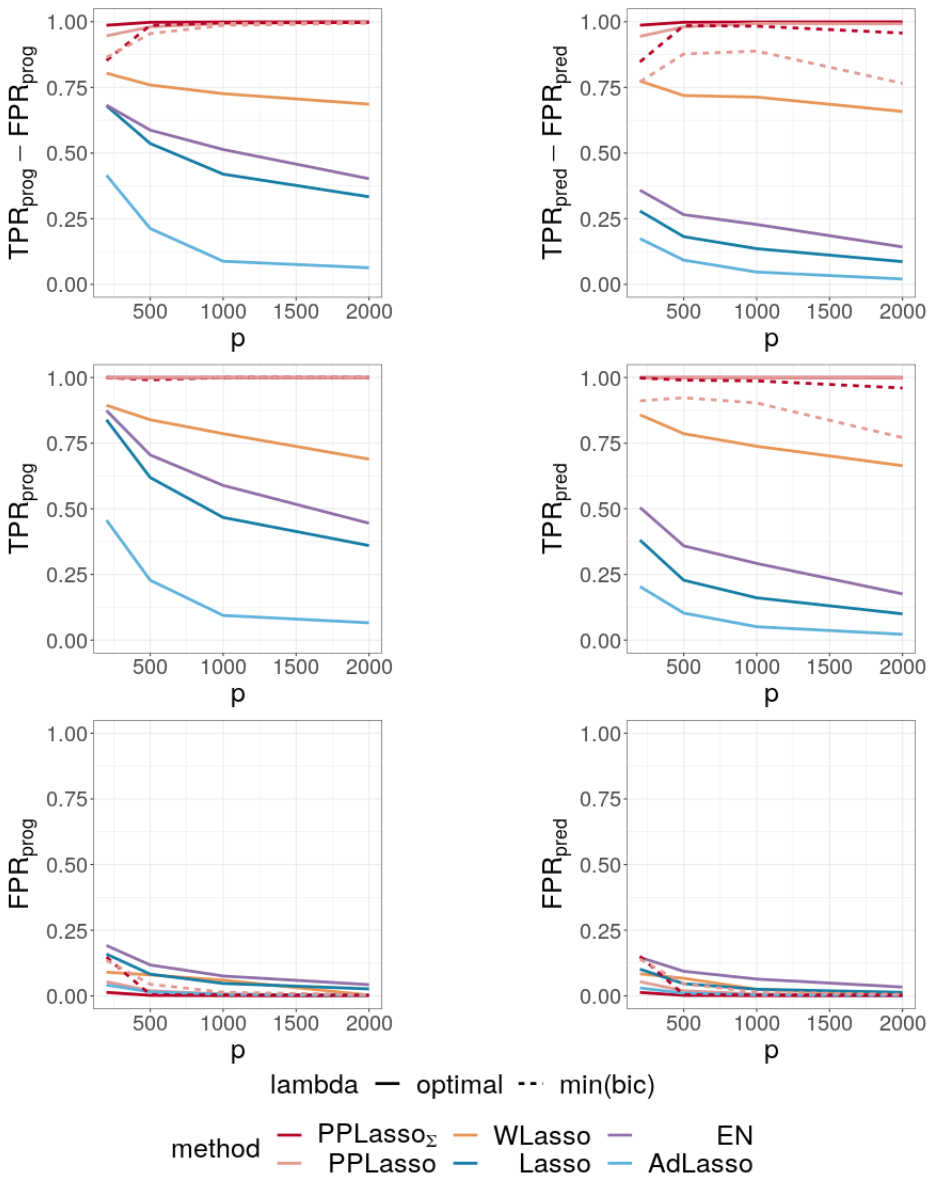

FIGURE S4. Average of (TPR-FPR) and the corresponding True Positive Rate (TPR) and False Positive Rate (FPR) for prognostic (left) and predictive (right) biomarkers (10 predictive biomarkers).

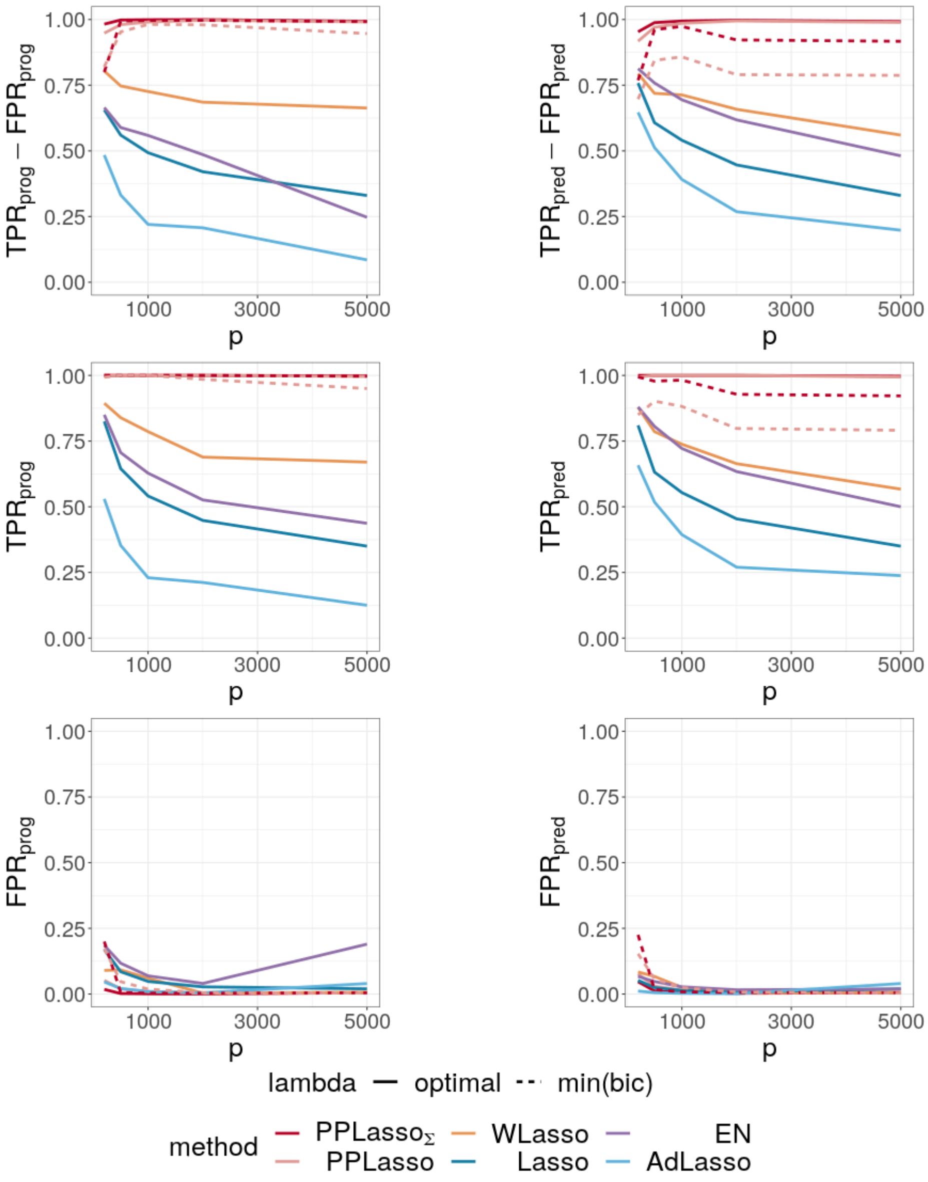

FIGURE S5. Average of (TPR-FPR) and the corresponding True Positive Rate (TPR) and False Positive Rate (FPR) for prognostic (left) and predictive (right) biomarkers (with $p = 5000$).

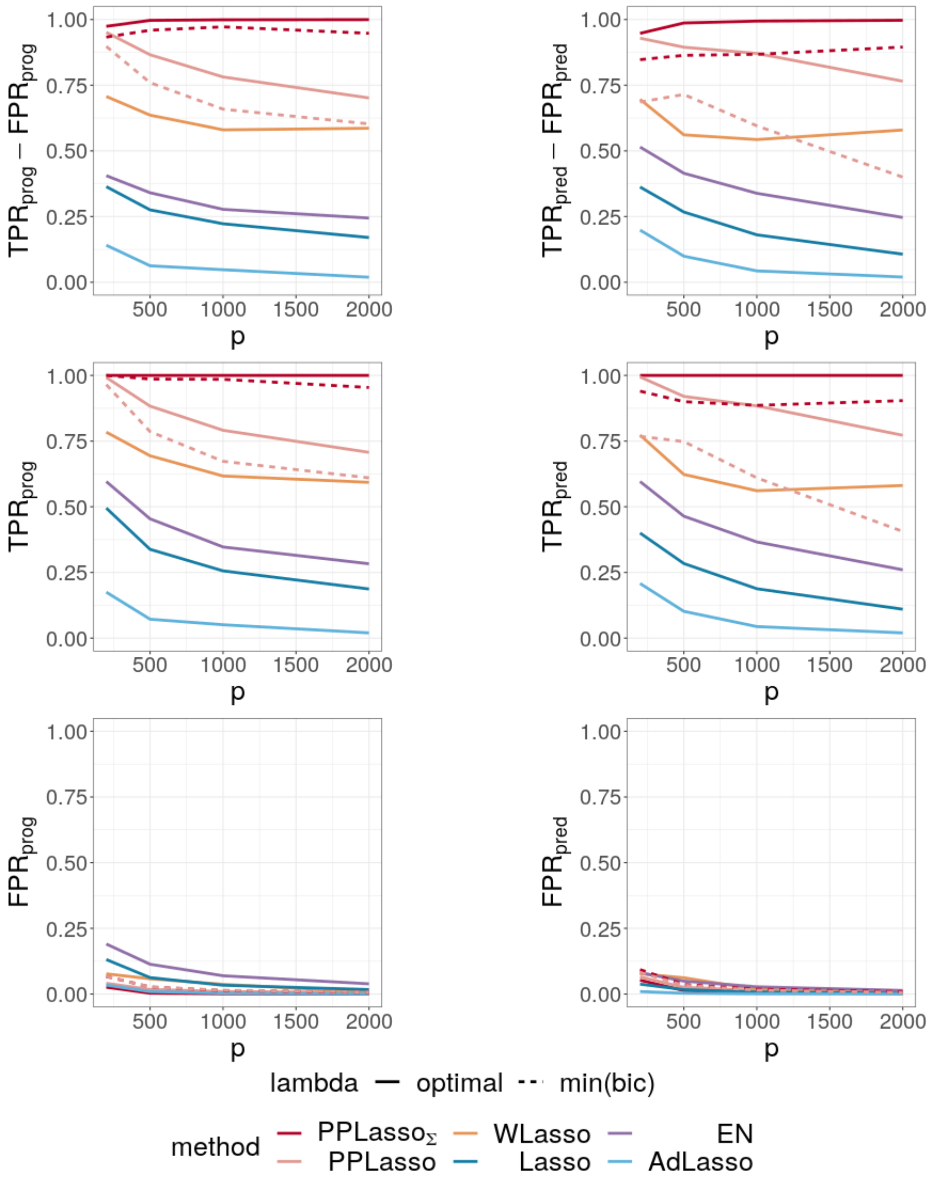

FIGURE S6. Average of (TPR-FPR) and the corresponding True Positive Rate (TPR) and False Positive Rate (FPR) for prognostic (left) and predictive (right) biomarkers ($n_1 = n_2 = 25$).

**Estimator**	**Hyperparameters**	**Empirical risk**
denseLinearShrinkEst	-	*102546*
sampleCovEst	-	102547
linearShrinkLWEst	-	103496
poetEst	lambda=0.1, k=2	104522
poetEst	lambda=0.2, k=2	105358
poetEst	lambda=0.1, k=1	105972
poetEst	lambda=0.2, k=1	108222
thresholdingEst	gamma=0.2	137798
thresholdingEst	gamma=0.4	186844

Table S2. Empirical risk of tested methods with different hyperparameters.

Pathway name	PPLasso	Lasso	EN	Ad.Lasso	WLasso
RUNX1 and FOXP3 control the development of regulatory T lymphocytes (Tregs)	✓	✓	✓	-	-
TNFR2 non-canonical NF-kB pathway	✓	✓	✓	-	-
TNFs bind their physiological receptors	✓	✓	✓	-	-
Activated PKN1 stimulates transcription of AR (androgen receptor) regulated genes KLK2 and KLK3	-	✓	-	-	-
Activation of anterior HOX genes in hindbrain development during early embryogenesis	-	✓	-	-	-
Activation of HOX genes during differentiation	-	✓	-	-	-
Amyloid fiber formation	-	✓	-	-	-
Assembly of the ORC complex at the origin of replication	-	✓	-	-	-
Assembly of the pre-replicative complex	-	✓	-	-	-
ATF4 activates genes in response to endoplasmic reticulum stress	-	✓	✓	✓	-
B-WICH complex positively regulates rRNA expression	-	✓	-	-	-
Base-Excision Repair, AP Site Formation	-	✓	-	-	-
Base Excision Repair	-	✓	-	-	-
Cellular responses to stimuli	-	✓	-	-	-
Cellular responses to stress	-	✓	-	-	-
Cellular Senescence	-	✓	-	-	-
Chromosome Maintenance	-	✓	-	-	-
Cleavage of the damaged purine	-	✓	-	-	-
Cleavage of the damaged pyrimidine	-	✓	-	-	-
Condensation of Prophase Chromosomes	-	✓	-	-	-
Cytokine Signaling in Immune system	-	✓	-	-	-
Defective pyroptosis	-	✓	-	-	-
Deposition of new CENPA-containing nucleosomes at the centromere	-	✓	-	-	-
Depurination	-	✓	-	-	-
Depyrimidination	-	✓	-	-	-
Diseases of programmed cell death	-	✓	-	-	-
DNA Damage/Telomere Stress Induced Senescence	-	✓	-	-	-
DNA methylation	-	✓	-	-	-
DNA Replication	-	✓	-	-	-
DNA Replication Pre-Initiation	-	✓	-	-	-
Epigenetic regulation of gene expression	-	✓	-	-	-
ERCC6 (CSB) and EHMT2 (G9a) positively regulate rRNA expression	-	✓	-	-	-
Estrogen-dependent gene expression	-	✓	-	-	-
Formation of the beta-catenin: TCF transactivating complex	-	✓	-	-	-
Gene Silencing by RNA	-	✓	-	-	-
HATs acetylate histones	-	✓	-	-	-
HCMV Late Events	-	✓	-	-	-

HDACs deacetylate histones	-	✓	-	-	-
Inhibition of DNA recombination at telomere	-	✓	-	-	-
Interleukin-10 signaling	-	✓	✓	✓	-
IRF3 mediated activation of type 1 IFN	-	-	✓	✓	-
Meiosis	-	✓	-	-	-
Meiotic recombination	-	✓	-	-	-
Meiotic synapsis	-	✓	-	-	-
Mitotic Prophase	-	✓	-	-	-
Negative epigenetic regulation of rRNA expression	-	✓	-	-	-
NoRC negatively regulates rRNA expression	-	✓	-	-	-
Nucleosome assembly	-	✓	-	-	-
Oxidative Stress Induced Senescence	-	✓	-	-	-
Packaging Of Telomere Ends	-	✓	-	-	-
PERK regulates gene expression	-	✓	✓	✓	-
Positive epigenetic regulation of rRNA expression	-	✓	-	-	-
PRC2 methylates histones and DNA	-	✓	-	-	-
Pre-NOTCH Expression and Processing	-	✓	-	-	-
Pre-NOTCH Transcription and Translation	-	✓	-	-	-
Recognition and association of DNA glycosylase with site containing an affected purine	-	✓	-	-	-
Recognition and association of DNA glycosylase with site containing an affected pyrimidine	-	✓	-	-	-
Reproduction	-	✓	-	-	-
RHO GTPases activate PKNs	-	✓	-	-	-
RMTs methylate histone arginines	-	✓	-	-	-
RNA Polymerase I Promoter Clearance	-	✓	-	-	-
RNA Polymerase I Promoter Escape	-	✓	-	-	-
RNA Polymerase I Promoter Opening	-	✓	-	-	-
RNA Polymerase I Transcription	-	✓	-	-	-
RUNX1 regulates genes involved in megakaryocyte differentiation and platelet function	-	✓	-	-	-
RUNX1 regulates transcription of genes involved in differentiation of HSCs	-	✓	-	-	-
Senescence-Associated Secretory Phenotype (SASP)	-	✓	✓	✓	-
SIRT1 negatively regulates rRNA expression	-	✓	-	-	-
Telomere Maintenance	-	✓	-	-	-
Transcriptional regulation by RUNX1	-	✓	-	-	-
Transcriptional regulation by small RNAs	-	✓	-	-	-
Transcriptional regulation of granulopoiesis	-	✓	-	-	-
Unfolded Protein Response (UPR)	-	✓	-	✓	-

Table S3: Over-representation pathway analysis using the REACTOME database and based on the prognostic genes identified by each method in the RV144 clinical trial transcriptomic data. Only pathways with p-values below 0.01 are displayed.

Pathway name	PPLasso	Lasso	EN	AdLasso	WLasso
IKK complex recruitment mediated by RIP1	✓	-	-	-	✓
NOD1/2 Signaling Pathway	✓	-	-	-	✓
Nucleotide-binding domain, leucine rich repeat containing receptor (NLR) signaling pathways	✓	-	-	-	✓
Regulated Necrosis	✓	-	-	-	✓
Regulation of necroptotic cell death	✓	-	-	-	✓
Regulation of TNFR1 signaling	✓	-	-	-	✓
RIPK1-mediated regulated necrosis	✓	-	-	-	✓
TICAM1, RIP1-mediated IKK complex recruitment	✓	-	-	-	✓
TNF receptor superfamily (TNFSF) members mediating non-canonical NF-kB pathway	✓	-	-	-	✓
TNF signaling	✓	-	-	-	✓
TNFR1-induced NFkappaB signaling pathway	✓	-	-	-	✓
TNFR2 non-canonical NF-kB pathway	✓	-	-	-	✓
Toll-like Receptor Cascades	✓	-	-	-	✓
Death Receptor Signalling	-	-	-	-	✓
Deubiquitination	-	-	-	-	✓
FGFR2 ligand binding and activation	-	-	-	-	✓
FGFR2b ligand binding and activation	-	-	-	-	✓
MyD88-independent TLR4 cascade	-	-	-	-	✓
Programmed Cell Death	-	-	-	-	✓
Scavenging by Class A Receptors	-	-	-	-	✓
Toll Like Receptor 3 (TLR3) Cascade	-	-	-	-	✓
Toll Like Receptor 4 (TLR4) Cascade	-	-	-	-	✓
TRIF(TICAM1)-mediated TLR4 signaling	-	-	-	-	✓
Ub-specific processing proteases	-	-	-	-	✓
Developmental Biology	-	-	-	✓	-
EGR2 and SOX10-mediated initiation of Schwann cell myelination	-	-	✓	✓	-
Gene and protein expression by JAK-STAT signaling after Interleukin-12 stimulation	-	✓	✓	✓	-
Interleukin-12 family signaling	-	✓	✓	✓	-
Interleukin-12 signaling	-	✓	✓	✓	-
Nervous system development	-	-	-	✓	-

TABLE S4. Over-representation pathway analysis using the REACTOME database and based on the predictive genes identified by each method in the RV144 clinical trial transcriptomic data. Only pathways with p-values below 0.01 are displayed.
